# The microbiota: a key regulator of health, productivity, and reproductive success in mammals

**DOI:** 10.3389/fmicb.2024.1480811

**Published:** 2024-11-05

**Authors:** Ibrar Muhammad Khan, Nourhan Nassar, Hua Chang, Samiullah Khan, Maoji Cheng, Zaigui Wang, Xun Xiang

**Affiliations:** ^1^College of Life Science, Anhui Agricultural University, Hefei, China; ^2^Department of Clinical Pathology, Faculty of Veterinary Medicine, Benha University, Moshtohor, Egypt; ^3^College of Veterinary Medicine, Yunnan Agricultural University, Kunming, China; ^4^The Scientific Observing and Experimental Station of Crop Pest in Guiyang, Ministry of Agriculture, Institute of Entomology, Guizhou University, Guiyang, China; ^5^Fisugarpeptide Biology Engineering Co. Ltd., Lu’an, China; ^6^College of Animal Science and Technology, Yunnan Agricultural University, Kunming, China

**Keywords:** healthy microbiota, ecological function, mammalian species, production traits, immune modulation, reproductive effeciency

## Abstract

The microbiota, intensely intertwined with mammalian physiology, significantly impacts health, productivity, and reproductive functions. The normal microbiota interacts with the host through the following key mechanisms: acting as a protective barrier against pathogens, maintain mucosal barrier integrity, assisting in nutrient metabolism, and modulating of the immune response. Therefore, supporting growth and development of host, and providing protection against pathogens and toxic substances. The microbiota significantly influences brain development and behavior, as demonstrated by comprehensive findings from controlled laboratory experiments and human clinical studies. The prospects suggested that gut microbiome influence neurodevelopmental processes, modulate stress responses, and affect cognitive function through the gut-brain axis. Microbiota in the gastrointestinal tract of farm animals break down and ferment the ingested feed into nutrients, utilize to produce meat and milk. Among the beneficial by-products of gut microbiota, short-chain fatty acids (SCFAs) are particularly noteworthy for their substantial role in disease prevention and the promotion of various productive aspects in mammals. The microbiota plays a pivotal role in the reproductive hormonal systems of mammals, boosting reproductive performance in both sexes and fostering the maternal–infant connection, thereby becoming a crucial factor in sustaining mammalian existence. The microbiota is a critical factor influencing reproductive success and production traits in mammals. A well-balanced microbiome improves nutrient absorption and metabolic efficiency, leading to better growth rates, increased milk production, and enhanced overall health. Additionally, it regulates key reproductive hormones like estrogen and progesterone, which are essential for successful conception and pregnancy. Understanding the role of gut microbiota offers valuable insights for optimizing breeding and improving production outcomes, contributing to advancements in agriculture and veterinary medicine. This study emphasizes the critical ecological roles of mammalian microbiota, highlighting their essential contributions to health, productivity, and reproductive success. By integrating human and veterinary perspectives, it demonstrates how microbial communities enhance immune function, metabolic processes, and hormonal regulation across species, offering insights that benefit both clinical and agricultural advancements.

## Graphical abstract

The graphical abstract generated with BioRender.com.
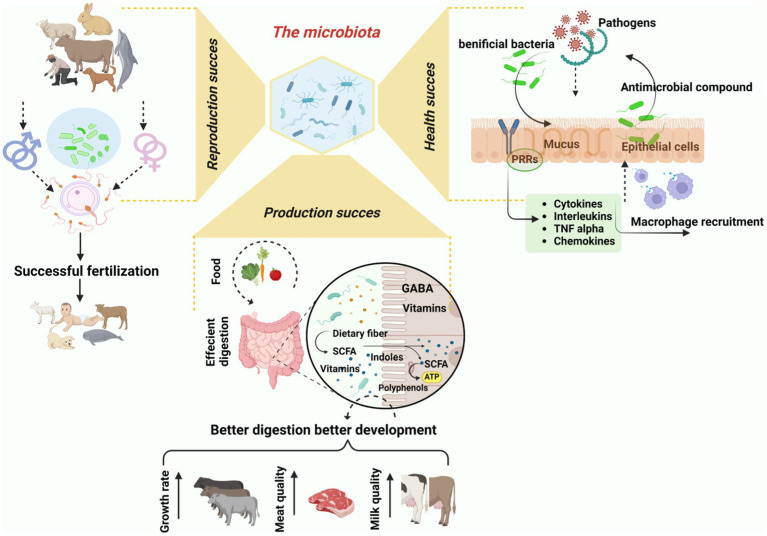

## 1 Introduction

The gut microbiota refers to the diverse community of microorganisms residing in the gastrointestinal tract. In contrast, the gut microbiome includes both these microorganisms and their genetic material. This intricate system is closely linked to mammalian physiology and plays a crucial role in regulating health, productivity, and reproductive functions (Dieterich et al., 2018). The human microbiota, often termed “the invisible organ,” contributes over 150 times more genetic material than the human genome (Dewi et al., 2023). The composition of the microbiota varies greatly depending on its anatomical location, shaped by factors such as pH, oxygen levels, nutrient availability, and host immune responses (Lloyd-Price et al., 2016). The gut microbiota is essential for neurodevelopmental processes such as blood–brain barrier formation, myelination, neurogenesis, microglial maturation, and the regulation of animal behavior. Consequently, it is believed to play a crucial role in the development and function of the nervous system (Sharon et al., 2016). Additionally, the gut microbiota influences ovarian dysfunction and insulin resistance in polycystic ovary syndrome (PCOS) and plays a role in the neuroendocrine regulation associated with depression and obesity in humans (Milaneschi et al., 2018; Qi et al., 2019). The gut microbiota actively regulates numerous host metabolic pathways, modulates signal transduction and inflammatory responses, and serves as a vital link between key tissues and organs, including the colon, liver, muscles, and brain (Nicholson et al., 2012). Fecal microbiota transplantation (FMT) shows promising potential in veterinary medicine. It has already been used to treat gastrointestinal disorders in dogs, and ongoing research is investigating its application for conditions like ruminal acidosis in cattle and colitis in horses (Niederwerder, 2018). Further research is needed to compare microbiomes across species to better understand the specific microbial patterns linking human and veterinary medicine.

Recent studies indicate that the gut microbiota is primarily composed of several key phyla: *Firmicutes*, *Bacteroidetes*, *Actinobacteria*, *Proteobacteria*, *Fusobacteria*, and *Verrucomicrobia*, with *Firmicutes* and *Bacteroidetes* being the dominant groups (Almeida et al., 2019; Hu et al., 2022). Emerging research has highlighted additional phyla like *Cyanobacteria and Tenericutes* that contribute to specific host interactions, emphasizing the ongoing evolution in our understanding of gut microbiota composition (Mishra et al., 2024). Further research is needed to compare microbiomes across species to better understand the specific microbial patterns linking human and veterinary medicine. Although common diseases in humans, livestock, and pets suggest shared microbial pathways, research on translating these findings across species remains limited. Firmicutes and Bacteroidetes dominate the microbiomes of many mammals, but the mechanisms governing these microbial interactions between species are still poorly understood (Laterza et al., 2016). Numerous research efforts have shed light on the crucial link between microbiota and fundamental biological functions in mammals. Recent developments, for instance, have demonstrated the significant role of human microbiota in the nutrients extraction, metabolic processes, and immune system function (Bouskra et al., 2008). Microbiota plays a crucial role in various biological processes, particularly in extracting energy and nutrients from food. This is due to its vast array of metabolic genes, which support diverse enzymes and biochemical pathways (Turnbaugh et al., 2006). In terms of the immune system, mammalian microbiota not only shields the host against foreign pathogens through the creation of antimicrobial agents but also plays a crucial role in the formation of the intestinal lining and the development of the immune system (Hou et al., 2022).

Advancements in omics-based technologies have transformed our comprehension of microbial communities associated with farm mammals and their health. The optimal functioning of the gastrointestinal tract (GIT) and its health are pivotal in influencing animal performance metrics such as body weight gain and the quality of milk and meat (Celi et al., 2017; Peixoto et al., 2021). Microbiota present in the gastrointestinal tracts of livestock and poultry break down and ferment the ingested feed, converting it into nutrients, are used to produce meat and milk (Liu et al., 2021). The symbiotic relationship between microbial communities and ruminant hosts enables the conversion of plant-based lignocellulosic biomass and non-protein nitrogen into volatile fatty acids and microbial protein. These substances are then available for the animal’s growth and maintenance (Lourenco and Welch, 2022).

Sex hormones like progesterone, estradiol, and testosterone contribute to the interaction between microorganisms and their hosts, playing crucial roles in various physiological processes. These include reproduction, cell differentiation, proliferation, programmed cell death (apoptosis), inflammation, metabolism, bodily equilibrium (homeostasis), and brain functionality (Qi et al., 2021b). Changes in the microbiota, especially within the gut, can have distinct effects on the reproductive hormonal system. Rectifying imbalances in the microbiome could result in enhanced reproductive health outcomes (Franasiak and Scott, 2015). The primary role of vaginal microbiota in humans and other mammals appears to be the enhancement of reproductive success. This is achieved by offering protection against infections and contributing to immunological robustness, both crucial for the health of the endometrium, fertility, successful embryo implantation, and the overall success of pregnancy (France et al., 2022; Golińska et al., 2021; Zhang et al., 2021). Certain metabolites present in the human vagina, such as glycerophospholipids and benzopyrene, have shown a positive association with the abundance of lactobacillus and are linked to a reduced incidence of repeated implantation failures (Garcia-Garcia et al., 2022). The microbiota plays a role in the development of male reproductive organs via the gut-brain axis, enhancing the production of androgens and safeguarding the immune tolerance of the testis. Androgens maintain the balance of regulatory T cells, curb the expansion of natural killer cells, and also fortify the blood-testis barrier to shield against harmful substances (Kabbesh et al., 2021). The microbiota facilitates the growth of Sertoli cells and their intercellular connections, thus guaranteeing the creation of seminiferous tubules and preserving the integrity of the surrounding microenvironment (Cai et al., 2022).

Grasping the biological roles of mammalian microbiota is essential for understanding its critical influence on health, productivity, and reproductive characteristics, making it a focal point in research areas due to its substantial impact on host biology. In this review, we present empirical evidence demonstrating that a balanced microbiota significantly enhances the health, productivity, and reproductive capabilities of mammals. The goal of this review is to elucidate the concealed capabilities and physiological impacts of microbiota across various mammalian species, laying a theoretical groundwork for future research into leveraging microorganisms for the well-being of both humans and animals.

## 2 Microbial ecology across various body regions of mammals

### 2.1 Skin microbiota

The body skin acts as a strong physical barrier to prevent physical trauma, environmental factors, and pathogenic invasion (Schmidt, 2020). Skin is the collective habitat of bacteria, viruses, fungi, and archaea, which is become a complex ecosystem and these microorganisms are essential to skin physiology and immunity. Interactions between skin microbiota and their hosts range from mutualistic to pathogenic relationships (Apprill et al., 2010). In contrast to the more diverse microbial communities found on haired skin, the mucosal surfaces of companion animals harbor less varied bacterial populations (Kamus et al., 2018). The teat skin microbiome has also received a lot of attention, especially in relation to the diversity of microbes found in raw milk. Major taxa found upon the teat surface skin included *Staphylococcus*, *Aerococcus*, *Pediococcus*, *Pantoea*, *Enterobacter*, *Enterococcus*, and *Proteobacteria* in addition to *Corynebacteriales*, *Atopobium*, *Clostridium*, *Bifidobacteriales*, *Lachnospiraceae*, and *Coriobacteria* (Verdier-Metz et al., 2012). Also, the skin microbiomes of aquatic mammals, like humpback whales, dolphins, and killer whales, have been examined as part of marine conservation efforts. For humpback whales in different ocean regions, *Psychrobacter* and *Tenacibaculum* were identified as core genera on their skin. The abundance of these genera varied depending on the metabolic states of the whales. The Offshore bottlenose dolphins demonstrated higher skin microbial diversity compared to their coastal counterparts, whose microbiomes were influenced by coastal run off (Russo et al., 2017). The captive dolphins displayed distinct microbiomes influenced by their respective environments, particularly food and air quality. These findings emphasize the importance of wild animals in future studies focused on improving the conservation of aquatic mammals affected by skin diseases (Cardona et al., 2018).

The human skin microbiome consists of different microorganisms, and they interact with surrounding environment, such as the existence of two distinct “cutotypes” on human skin has been discovered, each associated with unique patterns of microbial networks and host skin properties (Hoffmann, 2017). The four main bacterial phyla found on the skin are *Firmicutes* (24–34%), *Proteobacteria* (11–16%), *Actinobacteria* (36–51%), and *Bacteroidetes* (6–9%) (Byrd et al., 2018; McLoughlin et al., 2021). The dry regions (e.g., hypothenar palm and volar forearm) of the skin display diverse colonization patterns among the four phyla, showcasing the highest level of diversity (Lunjani et al., 2019; McLoughlin et al., 2021; Rozas et al., 2021). Increased levels of the phyla *Proteobacteria*, *Bacteroidetes*, *Spirochetes*, *Actinobacteria*, *Firmicutes*, *Ruminococcaceae*, *Aerococcaceae*, *Corynebacteriaceae*, and *Moraxellaceae* have been linked to healthy skin (Ariza et al., 2019; Krull et al., 2014) as shown in (Table 1).

**Table 1:** The relative abundance of skin microbiota in different mammalian species.

Species	Corresponding sample size	Body parts	Bacterial family	Geographic location	Biological sex	Study made by
*Bos taurus*	89 dairy cows	Punch biopsies of lesioned and healthy hooves	Proteobacteria, Tenericutes, Spirochaetes, Firmicutes, Bacteroidetes, Actinobacteria	New York, United States	89 females	Zinicola et al. (2015)
*Sus scrofa*	82 pigs sourced from Tibetan, Rongchang, and Qingyu breeds	Back skin near neck	Arthrobacter, Paenibacillus, Carnobacterium, Cellulomonadaceae, Xanthomonadaceae	Daocheng – eastern Tibetan plateau, Sichuan basin, China	Mix of boars and sows	Zeng et al. (2017)
*Myotis velifer*, *Myotis volans*, *Myotis californicus*, *Eptesicus fuscus*, *Tadarida brasiliensis*, *Corynorhinus townsendii*, *Antrozous pallidus*, *Parastrellus hesperus*, *Lasionycteris noctivagans*	186 bats from 13 species	Entire skin and furred region including ears, wings, uropatagia	Actinobacteria, Alphaproteobacteria, Gammaproteobacteria, Firmicutes	Arizona and New Mexico, United States	65 female and 95 males	Winter et al. (2017)
*Tursiops truncatus*	6 free-ranging bottlenose dolphins	Biospies	Lachnospiraceae, Gammaproteobacteria, Pseudomonas, Diaphorobacter, Acinetobacter, Acidovorax, Dechloromonas	Southern California	4 females, 2 males	Russo et al. (2017)
*Felis catus*	Healthy 11 and allergic 9	Healthy 12 skin spots, Allergic 6 skin spots	Alternaria and Cladosporium	Texas, United States	5 males and 6 females	Russo et al. (2017)
*Myodes glareolus*	157 wild bank voles	Dorsal thorax	Proteobacteria, Firmicutes, Actinobacteria, Bacteroidetes, Cyanobacteria	Ukraine: Kyiv and Chernobyl Exclusion Zone	63 males, 94 females	Lavrinienko et al. (2018)
*Bos taurus*	32 cattle	Hind limbs from abattoir	Steptococcus dysgalactiae, Treponema spp., *Klebsiella oxytoca*, *Fusobacterium necrophorum*, Pasteurella spp.	Copenhagen V, Denmark	Unidentified	Klitgaard et al. (2008)
*Equus ferus*	4 mares	Thorax and limb wounds had bandaged and unbandaged experimental groups	Planctomyetaceae, Acidobacteria, Fusobacteria, *Actinobacillus*	Montreal, Canada	4 mares	Kamus et al. (2018)
*Canis lupus familiaris*	12 healthy and 6 allergic dogs	12 skin sites (healthy), 4 skin sites (allergic)	Proteobacteria, Oxalobacteriaceae	Texas, United States	6 males, 6 females healthy; 4 males, 2 females allergic	Hoffmann (2017)
Pantroglodytes, *Gorilla gorilla*, Papio, *Macaca mulatta*	7 chimpanzees, 5 gorillas, 11 baboons, 2 rhesus macaques	Axillae	Firmicutes, Actinobacteria, Proteobacteria, Bacteroidetes	North Carolina zoo, United States.	Unknown	Council et al. (2016)
Tursiops truncates *Orcinus orca*	4 killer whales, 4 bottlenose dolphins	Dorsal, caudal, and pectoral fins; anal zone	Psychrobacter, Enhydrobacter, Staphylococcus, Sphingomonas	Antibes, France	2 males and 2 females per specices	Chiarello et al. (2017)
Lagenhorynchus obliquidens	4 Pacific white-sided dolphins	Periumbilicus skin	Pasteurellaceae, Peptostreptococcaceae, Fusobacteriaceae	Chicago, Illinois, United States	3 females, 1 male	Cardona et al. (2018)
*Megaptera novaeangliae*	89 humpback whales	Upper flank of dorsal spot	Psychrobacter, Moraxellaceae, Tenacibacterium, Flavobacterium	Western Antarctic Peninsula	Mix sex was collected	Bierlich et al. (2018)
*Megaptera novaeangliae*	56 humpback whales	Biopsy of upper flank near dorsal fin	Psychrobacter, Flavobacteria, Tenacibaculum, Gammaproteobacteria	North Pacific, North Atlantic, and South Pacific oceans	Not stated- no difference between sex observed	Apprill et al. (2014)

### 2.2 Respiratory tract microbiota

The respiratory tract including nose, nasopharynx, oropharynx, tonsils, hard plate, trachea, and lungs are contain a unique microbial community (McMullen et al., 2020). The following six *microbiome phyla*; *Proteobacteria*, *Bacteroidetes*, *Actinobacteria*, *Tenericutes*, *Fusobacteria*, and *Firmicutes* could be responsible for a healthy mammals respiratory tract system (Timsit et al., 2020); however, each phylum’s relative abundance and differences depending on the organ. The tonsils were colonized by *Fusobacteria*, while *Firmicutes* are widely distributed on the mouth’s floor and hard palate. *Proteobacteria* are predominant in the nose, nasopharynx, and oropharynx. *Streptococcus*, *Fusobacterium*, *Mycoplasma*, *Moraxella*, and *Streptomyces* are prevalent genus along the respiratory tract, with varying distributions: *Bibersteinia* is confined to the oropharynx, *Mycoplasma* dominated the tonsils, Streptococcus dominated the floor and hard plate of the mouth, and *Mycoplasma* dominated the trachea, lung, nostril, and nasopharynx (McMullen et al., 2020).

The human respiratory tract is consisting of niche-specific bacterial communities that live there from the nostrils to the lung alveoli. Respiratory pathogen colonization is likely inhibited by the respiratory tract’s microbiota, which is working as a gatekeeper. Additionally, the development and preservation of immune system and respiratory physiology homeostasis may be influenced by the respiratory microbiota. In relation to composition, the anterior nares are the most exposed to the outside world. They are lined with a keratinized squamous epithelium that resembles skin, containing serous and sebaceous glands. The latter secretes sebum, which enriches lipophilic skin colonizers such as *Propionibacterium* and *Staphylococcus* species and *Corynebacterium* spp. (Frank et al., 2010; Oh et al., 2014; Zhou et al., 2014). The anterior nares of human have also been exhibited the microbial hub including *Moraxella* spp., *Dolosigranulum* spp., and *Streptococcus* spp. that are frequently seen in other respiratory habitats (Pettigrew et al., 2012; Whelan et al., 2014; Wos-Oxley et al., 2016; Zhou et al., 2014). The stratified squamous epithelium covering the nasopharynx, which is situated deeper within the nasal cavity, is broken up by patches of respiratory epithelial cells. More species of *Moraxella*, *Staphylococcus*, and *Corynebacterium* are found in the nasopharynx’s bacterial communities, which are more diverse than those in the anterior parts and show significant overlap with the anterior nares. However, other bacteria, particularly *Haemophilus* spp., *Dolosigranulum* spp., and *Streptococcus* spp. (Biesbroek et al., 2014; Bosch et al., 2016; Teo et al., 2015), are more frequently encountered in the nasopharyngeal region. The oropharynx, characterized by a non-keratinized stratified squamous epithelium, harbors a broader array of bacterial populations compared to the nasopharynx41. These encompass species are streptococcal bacteria, *Neisseria* spp., *Rothia* spp., and anaerobes such as *Veillonella* spp., *Prevotella* spp., and *Leptotrichia* spp. (De Steenhuijsen Piters et al., 2016; Segata et al., 2012; Stearns et al., 2015).

### 2.3 Oral microbiota

The oral cavity, encompassing the tongue, saliva, gums, tooth surfaces, buccal mucosa, and other tissues, forms a complex network that provides a highly varied territory for microorganisms, predominantly bacteria (Kilian, 2018; Lu et al., 2019). Microorganisms inhabit both the solid surfaces of teeth and the soft tissues of the oral mucosa within the diverse niches present in the mouth, creating an exceptionally intricate ecosystem. Apart from serving as the starting point for digestion, maintaining the health of the oral microbiome is essential for maintaining overall systemic health (Caselli et al., 2020). Research indicates that once children acquire their first colonizing microorganisms, the diversity of their oral microbiome expands significantly (Gomez-Arango et al., 2016). Through various bidirectional communication and regulatory mechanisms, such as microbes in the gut or mouth, work together to maintain a homeostatic balance throughout an individual’s lifetime. Conversely, dysbiosis of the oral microbiota can contribute to the development of infectious diseases such as oral candidiasis, periodontal disease, and caries (Lamont et al., 2018). Given the critical role of oral health in mammals, extensive research has been conducted on the oral microbiomes of humans, as well as companion and farm animals like cats, sheep, and dogs. According to a recent report, *Burkholderia*, *Planifilum*, *Gastranaerophilales*, *Arcobacter*, *Escherichia-Shigella*, and *Actinobacteria* are the predominant genera associated with a healthy oral cavity in cattle (Borsanelli et al., 2017). The predominant bacterial phyla in the donkey oral microbiome, including *Actinobacteria*, *Bacteroidetes*, *Firmicutes*, *Proteobacteria*, and *Spirochaetes*, shared some similarities with the oral microbiomes of humans and other animals, albeit with slight variations. *Firmicutes*, a common phylum, was observed a common opportunistic pathogen in horse subgingival plaque and probably had been associated with periodontitis in other animal species. *Proteobacteria*, the second-highest phylum, was also present, and further investigations may shed light on its potential role in donkey periodontal diseases (Zhu et al., 2020).

A study made by Sturgeon et al. (2013) on oral microbiota in dogs, a core microbiome was identified, particularly *Porphyromonas* spp., and the association attributed to microenvironments in the dogs’ oral cavities, promoting the growth of some organisms while inhibiting others. The oral microbiome in dogs displayed moderate uniformity, high diversity, and greater richness compared to the canine fecal microbiome. Another study noted that *Porphyromonas* and *Fusobacterium* were highly abundant, raising questions about their roles as supporting pathogens in dogs, particularly in dental disease (Sturgeon et al., 2013).

### 2.4 Gut microbiota

The gut microbiota is a highly complex and heterogeneous ecosystem, where obligate anaerobes are typically 2 to 3 times more abundant than facultative anaerobes and aerobes (Quaranta et al., 2019). The rumen is frequently characterized as a “black box” owing to the intricate diversity and complexity of its microbial ecosystem. The ruminal microbiota is recognized as a functional organ, consisting of trillions of microorganisms, with a collective metagenome that surpasses the host’s genome by several hundred-fold (Human Microbiome Project, 2012). These microbial genes regulate the host’s nutrition consumption and overall health via specialized metabolic pathways. As a result, the ruminal microbiota is closely connected to host feed digestion and metabolic activities. Numerous studies have shown that different groups of the ruminal microbiota have a considerable impact on feed efficiency, nitrogen digestibility, and methane production in ruminants (Schären et al., 2018). For instance, rumen methanogenic archaea primarily utilize the end products of fermentation pathways, such as hydrogen and carbon dioxide, to produce methane (CH₄) (Patra et al., 2017).

Compared to the reticulum, omasum, and abomasum, the adult rumen plays the most crucial role in the degradation of dietary organic matter due to its diverse microbial population. Rumen microbes ferment dietary carbohydrates into volatile fatty acids (VFAs), which supply up to 80% of the total energy needed by ruminants (Liu et al., 2021). Some rumen microbes also synthesize their own proteins for growth, known as microbial crude protein (MCP), by utilizing energy and nitrogen derived from the feed. Once produced, MCP is digested in the small intestine and absorbed by the host, thereby contributing significantly to the host’s overall nutrition and health (Seshadri et al., 2018). *Bacteroidetes* is the most prevalent phylum in the rumen, and following by phylum Firmicutes. Moreover, the genera *Dialister*, *Succiniclasticum*, *Ruminococcus*, *Butyrivibrio*, and *Mitsuokella* collectively reported for over 1% of all bacterial genera present in the rumen (Myer et al., 2017). Numerous immune, metabolic, and nutrient absorption processes are essential to the host’s survival which mediated by the gut microbiota (Manus et al., 2017; O’Hara et al., 2020).

In non-ruminant animals such as pigs, horses, and humans, the gut microbiota is critical to a variety of physiological activities such as digestion, immunological regulation, and overall health. The microbiota is mostly found in the hindgut and ferments undigested dietary components such as carbohydrates, creating short-chain fatty acids (SCFAs) such as acetate, propionate, and butyrate, which are important energy sources for the host. Butyrate, for example, is particularly important in equine gut health because it promotes epithelial cell development and intestinal integrity (Koh et al., 2016). In pigs, the microbiota aids in food absorption by breaking down complex polysaccharides, proteins, and lipids, as well as generating critical vitamins including vitamin K and B vitamins, which contribute to the host’s nutritional status (Rook and Brunet, 2005). Furthermore, an imbalance in the microbiota, known as dysbiosis, has been linked to metabolic disorders such as obesity and insulin resistance, particularly in non-ruminant omnivores like humans and pigs, underscoring the microbiota’s role in energy metabolism and disease prevention (Cani and Delzenne, 2009). Altogether, the gut microbiota in non-ruminants is integral to maintaining health, regulating metabolism, and preventing disease. In human, the gut microbiota is predominantly composed of *Firmicutes* and *Bacteroidetes*, accounting for over 90% of the population. Phyla, such as *Actinobacteria*, *Proteobacteria*, *Fusobacteria*, and *Verrucomicrobia*, play a lesser role. In addition, *Spirochetes* and *Lentisphaerae* are present in smaller quantities. The gut microbiota also hosts various other microorganisms, including archaea, yeasts, fungi, viruses, and protozoa, although their composition remains uncertain (Carding et al., 2015).

#### 2.4.1 Small intestinal microbiota

Nonetheless, the role of the mammalian small intestinal microbiota in mediating the interactions between microbes and food is not yet fully understood. The host’s ability to adjust the dietary lipid variations depends on small intestine bacteria, which regulate the gut epithelial mechanisms involved in their digestion (Martinez-Guryn et al., 2018). The small intestine, which consists of the duodenum, jejunum, and ileum, serves as the primary site for nutrient absorption. Notably, it efficiently absorbs proteins and carbohydrates from the ingested food. Furthermore, within these intestinal compartments, intricate microbial ecosystems play essential roles in processes such as fermentation, vitamin synthesis, and immune modulation (O’Hara et al., 2020). It’s interesting to note that, exception of the jejunum, where proteobacteria predominated, the phylum *Firmicutes* dominated all other parts of the gastrointestinal tract in cattle. The jejunum enriched in *Acetitomaculum*, *Lachnospiraceae*, and *Ruminococcus*, whereas *Enterobacteriaceae* were highly abundant in the small intestine (Mao et al., 2015). The Firmicutes phylum had a sharp increase in relative abundance, reaching up to 80% of relative abundance, while the phylum Bacteroidetes significantly decreased (0.4:1.1%) in comparison to the rumen. There have also been published studies using low abundance phyla of *Proteobacteria* (0.8:5.8%), *Actinobacteria* (6:13%), and *Tenericutes* (0.4:4%). In addition, several other genera that are important for the small intestine are *Butyrivibrio*, *Ruminococcus*, *Mogibacterium*, *Mitsuokella*, *Propionibacterium*, *Lactobacillus*, and *Bulleidia* (Myer et al., 2017).

#### 2.4.2 Large intestinal microbiota

The large intestine plays a vital role in absorption of water, vitamins, electrolytes, and other nutrients (Scarpellini et al., 2015). Distinct sections of the large intestine exhibit varying microbial richness and abundance in their respective microbiota. In the cecum, *Firmicutes* emerge as the predominant phylum, constituting on 70–81% of all phyla, while *Bacteroidetes* comprise the remaining 18–26%. There have also been reported of *Actinobacteria*, *Tenericutes*, and *Spirochetes* in the cecum. Moreover, the most prevalent genera in the cecum have been found to be *Prevotella*, *Coprococcus*, *Dorea*, *Ruminococcus*, *Blautia*, *Turicibacter*, *Clostridium*, and *Oscillospira* (Myer et al., 2017) and they were the most prevalent genera (Myer et al., 2017; Myer et al., 2015). In a similar vein, the phylum *Firmicutes* has also taken control of the rectum. In addition, *Roseburia*, *Osillospira*, *Clostridium*, *Bacteroides*, *Succinivibrio*, *Ruminococcus*, *Prevotella*, *Blautia*, *Turicibacter* and *Coprococcus* were the genera that dominated the rectum (Durso et al., 2017).

### 2.5 Genital tract microbiota

**Figure 1:**
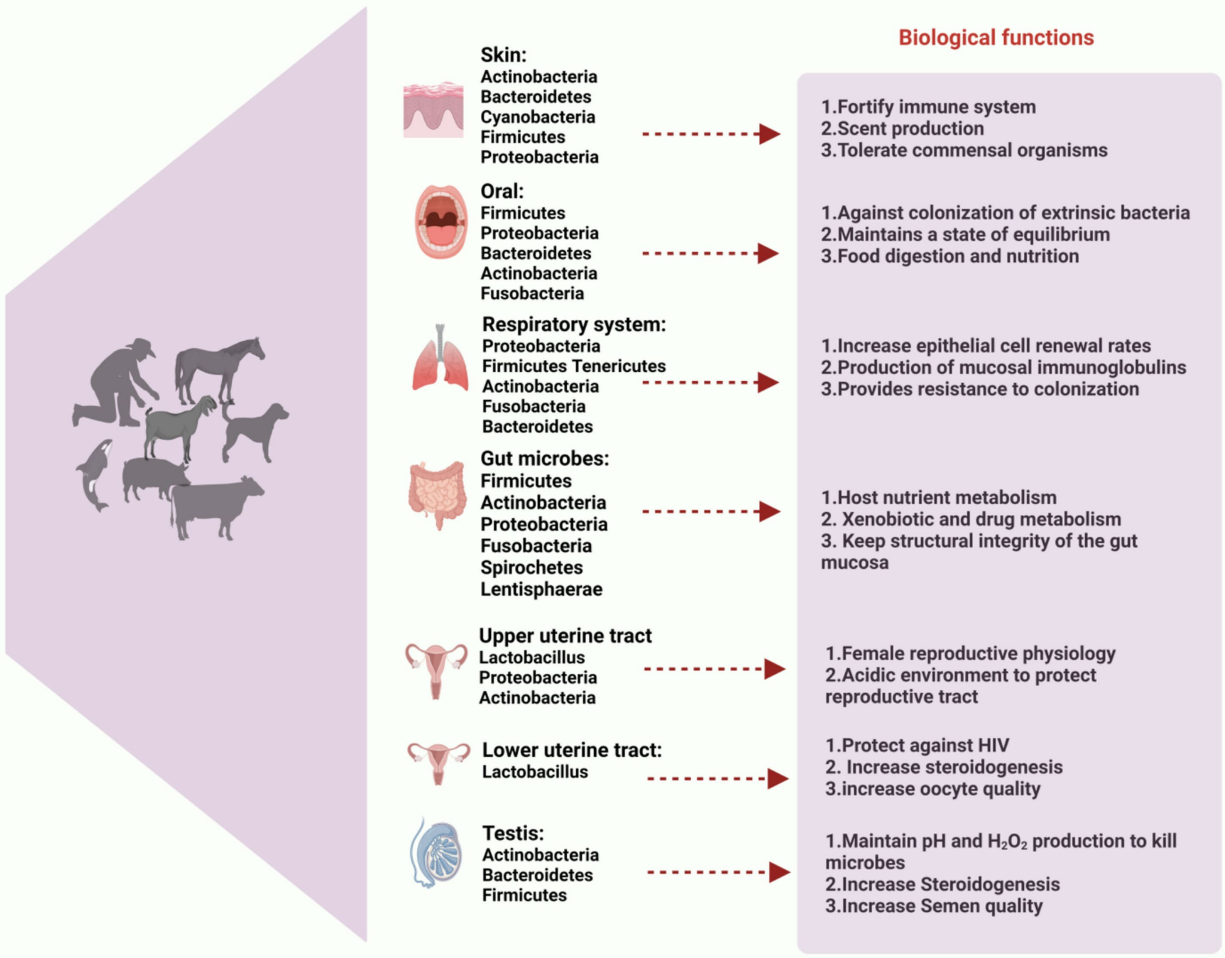
Composition of microbiota in different body organs. Predominant bacterial genera of skin, oral cavity, gut, respiratory tract, upper and lower uterine tract and testis are described and its emerging and biological role to health of mammals. This figure highlights the essential role of microbiota in maintaining overall health and homeostasis across various physiological systems. The figure created with BioRender.com.

Reproductive efficiency significantly influences health and homeostasis, as well as the overall productivity of mammals. From that vantage point, it becomes imperative to comprehend the microbiome of the reproductive tract (Manes et al., 2010). The oocyte’s ability to fertilize and its subsequent quality are directly influenced by the environment in which it grows. There has been inconsistent information about the presence of a microbiota in the reproductive tract. In human follicular fluid, some scientists have found cells and nucleic acids of bacteria (*Lactobacillus* spp., *Cutibacterium* spp., and *Actinomyces* spp.), but they have also documented changes between the right and left ovaries of the same host (Borges et al., 2020; Pelzer et al., 2011; Pelzer et al., 2013). However, regardless of the type of cyst and the presence or absence of endometriosis, a recent well-controlled study was unable to identify any particular microbiotas in ovarian cystic fluid (Oishi et al., 2022). The composition of the microbiota associated with follicular fluid has been successfully linked to pregnancy outcomes, even though its actual existence needs to be confirmed. Both healthy and infertile women showed a positive correlation between the presence of *Lactobacillus* spp., in the follicular fluid and the pregnancy rate following IVF and embryo transfer (Pelzer et al., 2013). Within the oviduct, crucial processes like fertilization, sperm capacitation, and early embryo development occur as part of a complicated signaling cascade. Limited information is available about the microorganisms that may inhabit or transit through the oviduct, despite the potential for interesting interactions between gametes and non-pathogenic oviductal bacteria. Semen typically contains a rich and diverse microbiota, which is important to note when discussing the male reproductive system (Cojkic et al., 2021; Farahani et al., 2020; Koziol et al., 2022; Wickware et al., 2020). Indeed, the bacterial communities found in oviducts appear to be similar to those found in semen (e.g., *Enterococcus* spp., *Cutibacterium* spp., and *Staphylococcus* spp.,) or the human vagina (*Lactobacillus* spp.,; Pelzer et al., 2018). Furthermore, it has been observed that the bacterial profiles exhibit variations in the fimbria and proximal oviduct (Brewster et al., 2022), the right and left oviducts, as well as the isthmus and ampulla (Pelzer et al., 2018). Thus far, no correlation has been found between these profiles and ovarian follicular or luteal status. In addition, menopause and hormone treatments can have an impact on the oviductal microbiota (Brewster et al., 2022). The endometrial immune system plays a crucial role in facilitating implantation and supporting fetal development, both of which are essential processes dependent on the uterine environment. According to a number of authors, the microbiota in the uterus appears to be distinct from that found in the vagina and is site-specific (Ichiyama et al., 2021; Lyman et al., 2019; Wang et al., 2021). The endometrial microbiome typically demonstrates higher bacterial variety and richness than the vagina and cervix in a wide range of animal taxa, including humans, giant pandas, dogs, domestic cattle, and horses (Diaz-Martínez et al., 2021; Ichiyama et al., 2021). The distinct bacterial genera inhabiting various body organs play pivotal roles in mammalian health, as illustrated in Figure 1, which highlights the predominant bacterial genera found in the skin, oral cavity, gut, respiratory tract, upper and lower uterine tracts, and testis, along with their emerging biological roles.

### 2.6 Cross talk between gut and reproductive microbiota

The gut microbiota plays a significant role in regulating reproductive hormones, which are essential for successful conception, gestation, and maternal–infant bonding. One of the key hormones influenced by gut microbiota is estrogen, which is vital for ovarian function and menstrual regulation. Studies have shown that the gut microbiota is responsible for the deconjugation of estrogens through enzymes like *β*-glucuronidase, which play a crucial role in regulating circulating estrogen levels. β-glucuronidase cleaves conjugated estrogens, converting them back into their active forms, which can then be reabsorbed into the bloodstream. This process is significant for reproductive health as elevated or imbalanced estrogen levels have been linked to various conditions, including endometriosis, polycystic ovarian syndrome (PCOS), and estrogen-dependent cancers such as breast cancer. Thus, understanding the activity of β-glucuronidase in the gut can provide insights into the modulation of estrogen-related health issues (Kumari et al., 2024). Dysbiosis can lead to reduced estrogen levels, which has been associated with reproductive disorders such as polycystic ovary syndrome (PCOS) and infertility (Baker et al., 2017). Additionally, gut microbiota influences other hormones crucial for reproductive health, such as progesterone and serotonin, both of which play roles in mood regulation and the establishment of pregnancy.

During pregnancy, the gut microbiota undergoes significant changes that prepare the mother for increased energy and nutritional demands. The alterations in microbial composition during the trimesters have been linked to the metabolic and immunological adaptations necessary for sustaining pregnancy and supporting fetal development (Koren et al., 2012; Nuriel-Ohayon et al., 2016). For instance, a study highlighted that specific bacterial genera increase during pregnancy, which may help modulate the immune response and reduce inflammation, supporting maternal health (Koren et al., 2012). Additionally, the microbiota may also play a role in maternal–infant bonding through the transfer of beneficial microbes during childbirth and breastfeeding, which can shape the infant’s developing microbiome. As, the way of delivery significantly impacts the infant’s initial microbial colonization, with vaginal births providing direct exposure to maternal microbiota that is essential for developing a robust immune system. In contrast, infants delivered via cesarean section often miss this critical microbial exposure, potentially affecting their health and their bonding with their mother (Rautava et al., 2012). Also, breastfeeding plays a crucial role in shaping the infant’s gut microbiota, as breast milk contains prebiotics and probiotics that foster the establishment of a healthy microbial community. This early microbial exposure is critical for establishing a healthy immune system and may enhance the emotional connection between mother and infant through the hormonal and biochemical signals modulated by these microbes (Bäckhed et al., 2015b; Pannaraj et al., 2017; Rautava, Luoto, et al., 2012).

Furthermore, the oral microbiota also changes during pregnancy, which has implications for maternal–infant bonding (Catassi et al., 2024). Research indicates that hormonal fluctuations during pregnancy can lead to changes in the oral microbiome, increasing the risk of conditions like gingivitis (Borgo et al., 2014; De Souza Abreu Alencar et al., 2016). This connection between oral health and hormonal changes suggests that a balanced microbiota could contribute to healthier pregnancies and possibly enhance maternal–infant bonding by reducing the risk of oral infections. Finally, emerging research points toward the potential of modifying the gut and oral microbiota to improve reproductive outcomes. Interventions aimed at restoring microbiota balance might help reduce inflammation and oxidative stress, enhancing fertility and maternal health. Future studies should focus on understanding these relationships and exploring therapeutic approaches to optimize microbiota health before and during pregnancy.

## 3 Mechanisms of interaction between host and microbiota

### 3.1 Host physiology

**Figure 2:**
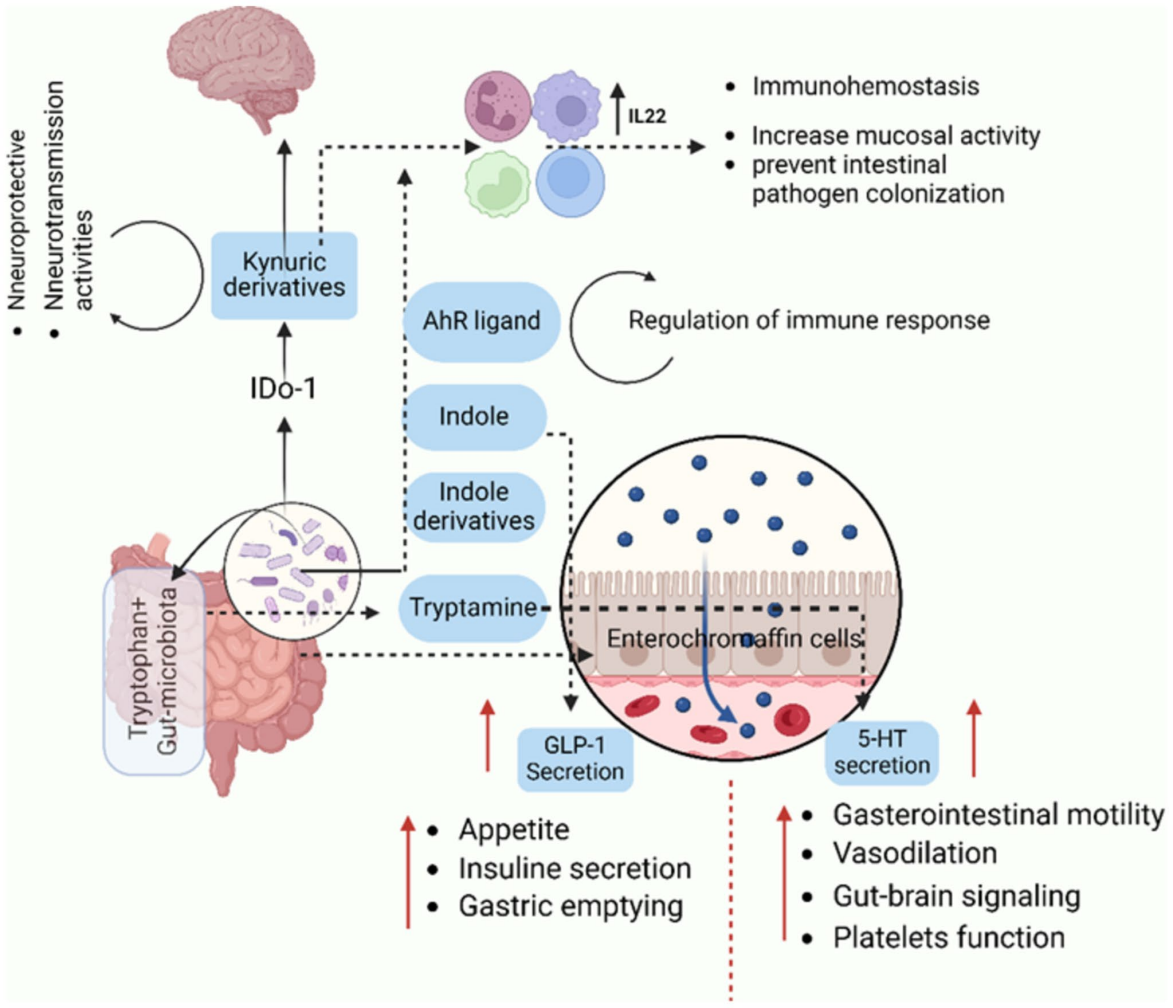
This sketch highlights how gut microbiota utilize tryptophan to produce metabolites that significantly influence physiological processes, including immune modulation, gut-brain communication, and appetite regulation. Understanding these pathways provides insights into the microbiota’s role in maintaining immune homeostasis, regulating gut physiology, and even affecting neurological and metabolic health. It offers potential therapeutic strategies targeting microbiota or tryptophan metabolism for disorders related to immunity, gut health, and brain function. The metabolism of tryptophan by gut microbiota involves several pathways that lead to the production of various metabolites. Gut microbiota can convert tryptophan into indole and its derivatives have been implicated in various physiological processes, including modulation of immune responses. Indole derivatives can activate the aryl hydrocarbon receptor (AhR) and it plays a role in immune modulation. Gut enterochromaffin cells can convert tryptophan into serotonin (5- HT), influencing gut physiology and other functions and also, influence the release of GLP-1 (glucagon-like peptide-1). Both GLP-1 and serotonin are involved in the regulation of appetite and satiety. Related to kynurenine Pathway, the kynurenine pathway can be used to metabolize tryptophan, producing a number of metabolites, including kynurenic acid, which is implicated in immunological modulation and has been linked to neurotransmission disorders. The figure created with BioRender.com.

As a barrier, the microbiota produces substances that improve mucus production, tight junctions within epithelia, wound healing, and stem cell proliferation. These elements guarantee that the contents of the intestine remain contained. Reduced barrier function allows microorganisms or their byproducts to leak into the body and improperly enter systemic circulation, frequently changing the inflammatory milieu (Zheng et al., 2020). Certain microbes produce surface metabolites or chemicals that can influence immune pathways, either promoting tolerance or triggering inflammation. The brain, heart, lymph nodes, or pancreas may all be affected systemically by these metabolites (Zheng et al., 2020), or they may act locally at sites where these microbes reside, such as the skin (Byrd et al., 2018), intestine, lung (Zhang et al., 2020), and mouth (Willis and Gabaldón, 2020). Additionally, the microbiota prevents the growth of pathogenic organisms that could cause or worsen disease by competing for nutrients or producing toxic and harmful metabolites (Ducarmon et al., 2019). Microbes that reside in different tissues have the capacity to produce molecules which have an immediate effect on the growth and functionality of cells (Burns and Guillemin, 2017). It is commonly discovered that microbial products affect host processes components of the outer membrane. The cell walls and outer membranes of microbes contain some of the most widely known elements and used for communication by the organisms. These communication molecules are among the most prevalent microbial products in the gut and frequently come into direct contact with host tissues. Peptidoglycan (PGN), for example, is a common component of all bacterial membranes and triggers a variety of immune signaling cascades (Zheng et al., 2020). Similarly, gram-negative bacteria’s cell wall contains a significant amount of lipopolysaccharide (LPS), which is also a strong systemic immune activator (Zheng et al., 2020). Highly immunostimulatory flagellins are also widely expressed in many different bacterial taxa. However, these compounds can also encourage immunological development and tolerance. For instance, balancing the immune cell populations in the gut is facilitated by the capsular polysaccharide polysaccharide A (PSA), which is presents by the commensal *B. fragillis*. These relatively common molecules are recognized by Toll-like receptors on various host tissues, along with numerous others and they alter host physiology both locally and systemically (Zheng et al., 2020). Numerous metabolites are produced by a diverse and healthy microbiota, and these metabolites have a variety of effects on host signaling pathways. As like the tryptophan metabolites, secondary bile acids, and SCFAs are the main types of metabolites with broad effects. Acetate, butyrate, and propionate are the SCFAs that are produced when dietary fiber ferments. SCFAs typically lead to positive host outcomes, including decreased rates of obesity and diabetes, increased tolerance to immunological stimuli, and even improved brain development (Cryan et al., 2019; Thomas and Jobin, 2019). Bile acids secreted by the liver into the gut can be broken down by certain bacteria. These secondary bile acids can affect the host in a number of ways, but they are most notable for their role in endocrine signaling that affects the liver-gut disease axis and metabolic homeostasis (Molinero et al., 2019). Indole, one of tryptophan’s metabolic byproducts, has been shown to have effects on hormone secretion, neurotransmitter expression, inflammation, and barrier function as in (Figure 2; Zheng et al., 2020).

### 3.2 Host immune system modulation

**Figure 3:**
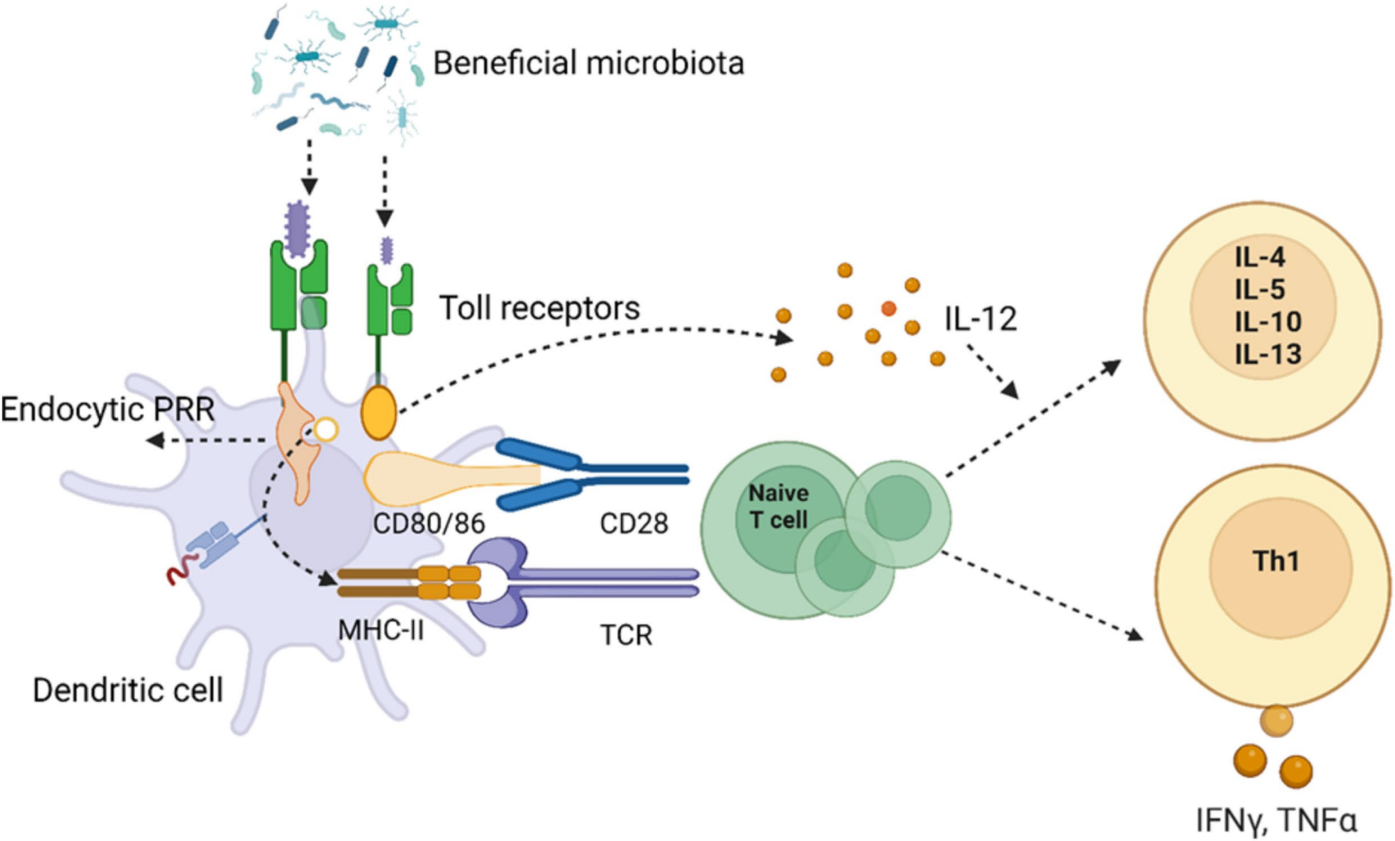
The sketch illustrates the intricate interaction between beneficial microbiota and the host immune system through Toll-like receptors (TLRs) on dendritic cells. The binding of microbial components to TLRs triggers a cascade of immune responses. Pattern recognition receptors (PRRs), such as endocytic PRRs, recognize microbial molecules, leading to the activation of dendritic cells. This activation involves upregulation of co-stimulatory molecules like CD80/86, which interact with CD28 on naive T cells through the major histocompatibility complex II (MHC-II) and T cell receptor (TCR) interactions. The dendritic cells release interleukin-12 (IL-12), a key cytokine that promotes the differentiation of naive T cells into T helper cells. Depending on the cytokine environment, T cells differentiate into various subtypes, including Th1 cells, which produce pro-inflammatory cytokines like interferon-gamma (IFN-γ) and tumor necrosis factor-alpha (TNF-*α*), driving cell-mediated immunity. Alternatively, T cells may differentiate into other subtypes producing anti-inflammatory cytokines such as IL-4, IL-5, IL-10, and IL-13, which modulate immune responses. This mechanism underlines the potential for modulating gut microbiota to influence systemic immunity and the potential therapeutic implications for inflammatory diseases. Created with BioRender.com

The immune response of host, which influences the susceptibility to disease, is significantly regulated by gut microbes and their metabolites (Hou et al., 2022). This happens via regular mechanical ways: Like, epithelial cells produce a number of anti- microbial proteins (AMP) and these peptides belong to the defensins, cathelicidins, and histamins families (Henrick et al., 2021). Secondly, IgA secreted by B-cells or plasma cells and recognize the microbial entry to the host (Al Nabhani et al., 2019; Chin et al., 2021). T-cells, also modulate the immune system like B-cells and become the part of the adaptive immune system, hence educated immune cells during early development to recognize self-antigens (Wang et al., 2019). The interplay between the gut microbiota and the immune system is essential for preserving host health, as the mucosal immune system acts as the primary defense against invasive gut microorganisms. Immune response elements such as tight junction proteins, antibacterial proteins, and a dense layer of mucus classify the mucosal surfaces. Innate immune cells in the gut develop tolerance to commensal bacteria by recognizing invasive pathogens and preventing their passage from the gut lumen into blood circulation (Wang et al., 2019). Upon breaching the epithelial barrier, invading bacteria and pathogen-associated molecular patterns (PAMPs), such as LPS, which swiftly reconstitute the inner mucous layer (McGuckin et al., 2011). PAMPs can induce the production of mucin from goblet cells, and they can also activate Toll-like receptors (TLRs) on neutrophils and macrophages, triggering innate immune responses (Mogensen, 2009). Additionally, commensal bacteria can activate TLRs, guiding the innate immune system to differentiate between pathogenic and commensal microbes by stimulating dendritic cells (DCs) through antigen presentation (Minarrieta et al., 2016) as presented in (Figure 3). Under normal conditions, mucosal innate immune cells, such as dendritic cells (DCs) and macrophages, engulf and eliminate invading microbes through phagocytosis (Levy et al., 2017). Furthermore, a recent discovery highlighted that the gut microbiota triggers the secretion of tumor necrosis factor (TNF) by monocytes and macrophages, facilitating the maturation of precursors type 1 conventional DCs (Köhler et al., 2020). In support of gut innate immunity, specialized epithelial cells, like goblet cells and Paneth cells, release various antimicrobial substances, including mucins, defensins, lysozyme, secretory phospholipase A2, and cathelicidins. These cells serve as supplementary immune cells alongside macrophages, neutrophils, and DCs (Johansson and Hansson, 2016). The interaction between the adaptive immune system and gut microbiota serves as a preventive measure against bacterial translocation and infection. This is demonstrated and observed that gut adaptive immune system is suppressed in germ-free mice, and the introduction of commensal bacteria can foster the development of mucosal lymphocytes, including cytotoxic CD8+ T cells and CD4+ T cells (Suzuki et al., 2010). The antigen-presenting cells, prime CD4+ T cells, and their signaling is crucial for both the primary and secondary phases of cytotoxic CD8+ T cell immunity (Bedoui et al., 2016). CD8+ T cells eliminate intracellular pathogens like *Salmonella* through antigen presentation mediated by dendritic cells (DCs) (Belz et al., 2005). The Transient Microbiota Depletion-boosted Immunization model (Becattini et al., 2020) offers a gateway to temporarily suppress microbiota-mediated colonization resistance, enabling the study of the role of tissue resident memory CD8+ T cells in preventing re-infection instances. Notably, Th17 cells induced by *Citrobacter* spp., exhibit pro-inflammatory characteristics, while Th17 cells stimulated by segmented filamentous bacteria (SFB) are non-inflammatory (Omenetti et al., 2019). Studies have revealed that germ-free mice lack Th17 cells, activated by specific microbes like SFB (Ivanov et al., 2009) and other commensal bacteria (Tan et al., 2016). It is uncovered that cytokine signals, including IL-6, guide SFB-mediated IL-17 stimulation (Sano et al., 2021). In addition, the gut microbiome can impact Th17 responses; as investigation suggests that α2,6-sialyl ligands regulate microbiome-dependent Th17 inflammation, and α2,6-sialyltransferase deficiency triggers mucosal Th17 responses (Irons et al., 2022). Within the gastrointestinal tract (GIT), regulatory T cells (Treg) constitute an additional category of adaptive immune cells that play a role in immune tolerance. Natural Treg cells are generated in the thymus during early life through the action of an autoimmune regulator, promoting self-tolerance (Malchow et al., 2016). Subsequently, peripheral or inducible Treg production is initiated through exposure to diet and the microbiota (Ramanan et al., 2020). The gut microbiota can stimulate Treg cells in various ways. For instance, to maintain immune tolerance in the intestine, ILCs can opt for RORγt + Treg cells that specifically target the microbiota, inhibiting the proliferation of Th17 cells (Lyu et al., 2022). Immunological responses mediated by RORγt + Treg cells can also be induced by *Helicobacter* spp. (Chai et al., 2017) and *A. muciniphila* (Liu et al., 2022).

### 3.3 Colonization resistance and pathogen inhibition

The microbiota prevents pathogens from invading the intestinal ecosystem, a phenomenon known as colonization resistance. The gut microbiota consists of multiple commensal bacteria that may provide colonization resistance through multiple parallel mechanisms, including food struggle, niche exclusion, competitive metabolic interactions, and initiation of host immune response against the harmful bacteria (Pickard et al., 2017). In addition to direct colonization resistance, symbiodinium can modify the intestinal microenvironment to stop the colonization of pathogens. The gut microbiota competed for nutrients, and formed cross-feeding patterns and substrate preferences during evolution to maximize the utilization of existing nutrient. Under steady-state conditions, exogenous strains are unlikely to find an uncompetitive ecological niche and will be forced to compete for nutrients with the normal microbiota in the gut (Sorbara and Pamer, 2019).

Bacteriocins derived from microorganisms have been identified to be active against both Gram-positive and Gram-negative pathogens, typically active against bacteria that are closely related, while others may be more broadly active (Sorbara and Pamer, 2019). There are many kinds of bacteriocins, such as those produced by lactic acid bacteria metabolism can inhibit many bacteria, fungi and viruses, and their antibacterial mechanism of action include destruction of cell membrane, forming transmembrane ion channel and intracellular action of bacteriocins, and interfering with the normal metabolism of bacteria (Pato et al., 2022). The helical structure of Hcp, inner tube and VipA/B outer sheath provides sufficient penetration of the T6SS into the target cell membrane and cell wall (Liang et al., 2018). At present, T6SS is only found in gram-negative bacteria, more than half of the human intestinal Bacteroides genome and more than a quarter of the proteus genome contain T6SS gene, it has a strong bactericidal ability (Coyne et al., 2016). Through direct contact between cells by Type VI Secretion System (T6SS) and physical penetration transport cytotoxic secreted proteins to neighboring cells and eukaryotic cells, can provide colonization resistance to pathogenic bacteria (Burkinshaw et al., 2018). Indirect colonization resistance, SCFAs on the inhibition of bacterial virulence and replication: studies have shown that high concentration of SCFAs inhibit *enterobacteriaceae*, most SCFAs produced in the proximal colon, absorbed by the host to support intestinal epithelial cell metabolism, high concentration of SCFAs lead to intestinal lumen acidification, induced *enterobacteriaceae* bacteria acidification to inhibit its replication mode (Sorbara et al., 2019).

### 3.4 Intestinal M cells regulation and GIT protection

The gut microbiota is a diverse community of symbiotic bacteria that live in the gastrointestinal tracts of mammals. These bacteria, which are thought to number 40 trillion or more in humans, and the more numbers living in the colon part (Sender et al., 2016). The secretory IgA (SIgA) represent the hallmark of immune response at mucosal sites and contribute to homeostasis via a variety of mechanisms. SIgA antibodies are induced by postnatal exposure to commensal microbiota indicating that these antibodies play a role in sensing commensal microbes and limiting their overgrowth. SIgA antibodies also protect the host by binding to the surface of luminal microbes and toxins to prevent them from attaching to epithelial cells (Boyaka, 2017). IgA binds to the toxin and removes that produced by the harmful microbes, thus keeping the germs out of the intestinal lumen and preserving intestinal homeostasis (Bunker and Bendelac, 2018). The host mucosal immune system has evolved a technique to test the gut microbiota from the intestinal lumen in order to identify these bacteria as toxins. Mucosal tissues are involved in host adaptive immune responses both as effector and inductive sites. Important inductive sites in the intestine are Peyer’s patches (PPs) and other gut-associated lymphoid tissue (GALT) (Brandtzaeg et al., 2008; Suzuki et al., 2010). Antigen sampling is the transfer of antigenic material from the external environment across the epithelium to immune cells located beneath the epithelial layer, is the initial step while starting the antigen-specific mucosal immune responses (Schulz and Pabst, 2013). M “microfold” cells, which are specialized epithelial cells that effectively mediate antigen sampling, are part of the follicle-associated epithelium (FAE) that covers the lymphoid follicles of GALT. The lymphoid follicles of GALT are not reached by antigen or antigen-presenting cells via afferent lymphatics Gebert, 1997. Relatively, M cells offer one of the main routes for directly sampling of commensal enteric bacteria and other antigenic material in the intestinal lumen. Antigens can be quickly transported to dendritic cells that are closely linked to M cells in the subepithelial dome (SED). After processing, antigens are given to T cells, which aid in B-cell maturation, activation and production of IgA-producing cells. Therefore, the secretory IgA response in the intestine is initiated by M-cell-mediated antigen transcytosis (Kraehenbuhl and Neutra, 2000). Thus, the enteric microbiota become a significant element impacting M-cell differentiation. The M cells come into contact with antigens present in the gut lumen. This exposure triggers M cells to capture the antigens, transport them to dendritic cells, and ultimately initiate immune responses (Tahoun et al., 2012).

## 4 Nutritional contribution of microbiota in mammals

### 4.1 Feed conversion into nutrients

Together with the diverse microbial ecology, the enzyme activities in the liver and gut mucosa perform a wide variety of metabolic functions and crucial for the host’s digestion. As a result, the gut microbiota has a substantial impact on the biochemical composition of the diet and its implications for host health and disease (Rowland et al., 2018). The substrates that are not absorbed or digested in the upper GI tract can be fermented by microbiota in the colon. These substrates consist of foods like proteins, fats, carbohydrates, and other substances that are difficult for the body to digest because of their intricate molecular makeup (Yao et al., 2015). Gut microbes break down complex dietary components such as fibers, starches, and proteins that the host’s own enzymes cannot fully process. By fermenting these substrates, they release simpler compounds (e.g., short-chain fatty acids, amino acids, and vitamins). These breakdown products are more easily absorbed by the host’s intestinal cells, ensuring efficient nutrient uptake (Rowland et al., 2018). For instance, the rumen, which is regarded as a compartment for anaerobic and methanogenic fermentation, can exploit cellulolytic feeds to increase production, and residing microbiota considerably aiding in feedstuff breakdown (Morgavi et al., 2012). These microorganisms, including the bacteria, archaea, protozoa, and fungi, continuously ferment the food, also break down into constituent parts. These VFAs serve as energy sources for the ruminant, supporting growth, milk production, and overall vitality. Additionally, gut microbes participate in ammonia detoxification, preventing toxic buildup. The most significant pectinolytic species are *Lachnospira multiparus*, *Prevotella ruminicola*, and *Butyrivibrio fibrisolvens*; and these bacteria can break down pectin into oligogalacturonides, leading into significant amounts of acetate, a volatile fatty acid for ruminant metabolism (Duskova and Marounek, 2001).

#### 4.1.1 SCFA and nutrient metabolism to disease prevention

The large intestine microbiota mostly requires nutritional substrates that are not fully digested in the upper digestive tract. When the carbohydrates source becomes scarce, bacteria will seek alternative sources of energy, perhaps leading to the development of more toxic metabolites. However, saccharolytic bacterial fermentation produces metabolites that are generally beneficial (Boyd et al., 2013). The primary byproducts of bacterial fermentation of dietary carbohydrates are gasses and SCFAs. Short-chain fatty acids (SCFAs), primarily acetate, propionate, and butyrate, play essential roles in gut health and systemic disease prevention. Butyrate is crucial for maintaining colonocyte energy supply, enhancing gut barrier integrity, and modulating immune responses by promoting the differentiation of regulatory T cells (Tregs) and suppressing inflammation, thus helping to protect against conditions like inflammatory bowel disease (IBD) and colorectal cancer (Liu et al., 2018). Propionate contributes to metabolic health by inhibiting cholesterol synthesis and improving insulin sensitivity, thereby reducing the risk of metabolic disorders such as type 2 diabetes (Zhao et al., 2018). Also, it delivered to the liver, where it helps to gluconeogenesis while also providing energy to epithelial cells. Correspondingly, playing a significant role in satiety signaling due to its interaction with gut receptors, notably G protein-coupled receptors GPR 41 and GPR 43, also known as fatty acid receptors FFAR2 and FFAR3. This interaction may, initiate intestinal IGN (Brown et al., 2003; De Vadder et al., 2014; Karaki et al., 2008). Intestinal gluconeogenesis converts propionate to glucose, which reduces hepatic glucose production, obesity, and thus directly promotes energy homeostasis. Acetate, the most abundant SCFA, has been linked to appetite regulation and blood pressure control, with studies showing its beneficial effects on cardiovascular health (Yang et al., 2022). Acetate, the most common SCFA, is an essential cofactor and metabolite for the growth of other microorganisms. For instance, in the absence of acetate, *Faecalibacterium prausnitzii* requires acetate to grow in pure culture, as it lacks the ability to synthesize this compound independently (Duncan et al., 2004). Acetate is transported to the periphery of the human body, where it is used in lipogenesis and cholesterol metabolism. More recently, studies on mice have demonstrated that acetate is essential for central appetite control (Morgavi et al., 2012). A recent cohort study reported that higher circulating SCFA levels were associated with a 20% reduced risk of type 2 diabetes (Koh et al., 2018), while another study demonstrated that SCFAs reduced the severity of colitis by 30% in experimental models (Furusawa et al., 2013).

#### 4.1.2 Protein and vitamin synthesis

The colonic microbiota is a potent proteolytic agent that can break down ingested dietary protein as well as endogenous protein from host enzymes like mucin, and shed intestinal cells into shorter peptides, amino acids, as well as short and branched-chain fatty acids and gasses like ammonia, H_2_, CO_2_, and H_2_S (Macfarlane et al., 1992). Saccharolysis is predominant in the proximal colon, However, protein fermentation and pH levels rose in the transverse and distal colons. Increased amounts of phenol, indole derivatives resulting from amino acid fermentation, branched-chain fatty acids, and ammonia is linked to protein fermentation (Hylemon et al., 2018; Kalantar-Zadeh et al., 2019). Sulfate-reducing bacteria (SRB), which can convert sulfate compounds to H_2_S, produce minor gasses like H_2_S and other sulfur-containing gasses in trace amounts (Mutuyemungu et al., 2023). The amino acids taurine, methionine, and cysteine found in animal proteins, as well as other sulfated polysaccharides like carrageenan, would be the sources of sulfate in the colon (Rey et al., 2013). Research involving aseptic and conventional mice, as well as human volunteers, suggests that the gut microbiota possesses the ability to synthesize certain vitamins. These include vitamin K and various B group vitamins such as biotin, cobalamin, folates, nicotinic acid, pantothenic acid, pyridoxine, riboflavin, and thiamine (Hill, 1997).

### 4.2 Meat quality traits

Sustainable meat production is crucial to ensure its availability all across the world and people have access to healthy and high-quality protein. The Organization for Economic Cooperation and Development (OECD) and the Food and Agriculture Organization of the United Nations (FAO) predict that global meat output will grow by 2030 (OECD/FAO 2021). Over the last 10 years, vigorous artificial selection and high energy intake have enhanced daily body weight gain and reduced raising time in many commercial animals, but have accidentally resulted in worse meat quality. Animal gastrointestinal tracts are home for abundant and varied microbial community that is essential to immune system development, meat quality, pathogen elimination, and nutrient digestion and absorption (Chen et al., 2022). The gut microbiota, which is intimately related to host metabolism and health, has been dubbed the second set of the host genome (Noel et al., 2019). Research has indicated a strong correlation between fat metabolism and the gut microbiota (Kuno et al., 2018; Zierer et al., 2018). By sequencing the 16S rRNA gene in the intestinal microbiota of distinct pig gut segments and examining the correlation with meat quality traits (MQTs). Thus, the authors demonstrated that the traits linked to fat deposition in pigs were more significantly influenced by the microbiota of the cecum, colon, and jejunum (Chen et al., 2022). Additionally, a study conducted on castrated Holstein bull as reported by Whon et al. (2021) and examined their gut microbiota profile and MQTs, result suggested an increased extra and intramuscular fat (IMF) storage and a higher relative abundance of the family *Gastrostreptococcus*. Meat quality is complicated term influenced by a variety of elements, most notably customer preferences. Castrated male cattle harbor distinct ileal microbiota dominated by the family *Peptostreptococcaceae* and exhibit distinct serum and muscle amino acid profiles (i.e., highly abundant branched-chain amino acids), with increased extra- and intramuscular fat storage (Whon et al., 2021). According to Zheng et al. (2022), there is a direct relationship between genes associated with muscle metabolism, such as MYLPF, MSTN, ATP2A1, TNNT3, ACTN3, and MYL1, and gut microbial species *B. uniformis*, *B. vulgatus*, *R. inulinivorans*, *C. catus*, *F. prausnitzii*, and *E. rectale*; these species have a direct impact on meat quality. The butyrate-producing bacterium *Faecalibacterium* was linearly connected with the Angus breed, which is known for its high IMF. *Akkermansia*, a mucin-degrading bacterium known for regulating energy expenditure, was found to be more abundant in Brahman calves with lower levels of IMF (Fan et al., 2019). Pigs’ carcass configuration and meat quality characteristics were measured in order to estimate the microbiome’s heredity. The study revealed a strong positive microbiological correlation between various traits, specifically those associated with meat color and firmness score (Khanal, 2019). Additionally, there were variations in the microbial community’s diversity and composition among the various swine breeds. Notably, the Duroc breed, known for its superior meat quality, tenderness, increased flavor, and palatability, had a different microbial community when compared to other breeds (Pajarillo et al., 2014; Pajarillo et al., 2015).

Another, the research demonstrated that the gut flora affects the deposition of intramuscular fat. It is probable that the gut microbiota primarily affects adipose formation through distinct adipogenic pathways (Krause et al., 2020). Furthermore, it was found that fatty and lean-type pigs differed in the abundances of colonic bacteria and bacterial metabolites (Jiang et al., 2016). Similarly, other research revealed a correlation between higher IMF content in pigs and an elevated *Firmicutes* to Bacteroidetes ratio and increased genus Romboutsia abundance in colonic samples (Wu et al., 2021).

### 4.3 Milk production

Most studies on milk microbiota have primarily focused on mammalian species such as humans (Fitzstevens et al., 2016) as well as domestic animals including cows, goats, sheep, and donkeys (Addis et al., 2016; Falentin et al., 2016). Recent advances in biotechnology enable microbial production of specific Human Milk Oligosaccharides (HMOs), (e.g., 2′-fucosyllactose, lacto-N-neotetraose, 3-fucosyllactose and lacto-N-tetraose). These techniques like whole-cell catalysis and fermentation facilitate efficient biosynthesis of these HMOs (Deng et al., 2020). Microbes also help to synthesize proteins such as caseins and whey protein, and microbial enzymes are involved in lipid metabolism, lactose breakdown, and other processes (Deng et al., 2020). A study found that the efficiency of milk production in cows is linked to their gut microbiome. Less milk-producing cows have undigested nutrients in their large intestine, requiring more beneficial bacteria to breakdown these nutrients, whereas the efficient cows having normal gut microbiota obtained more energy from the undigested nutrients. When Holstein cows eat high-forage diets, their rumen microbiome has more enzymes for breaking down plant components. The high milking cows gut have more fibrolytic bacteria with enzymes, while less producer cows have other class of bacteria associated with lower efficiency (Monteiro et al., 2022).

### 4.4 Gastric development in weaning mammals

According to this study, dairy calves are born with an underdeveloped GIT and a non-functioning rumen. Compared to adult animal, the rumen has lower proportions and is devoid of some important functional elements, such as the villi in the rumen wall, which are crucial for nutritional absorption (Meale et al., 2017). During the first 3 weeks of life, milk is the primary food source, entering the abomasum through the esophageal groove rather than the rumen. The formation and expansion of the rumen microbiota, particularly starch-degrading bacteria, is triggered by the highly appetizing starting feed which is fermentable into carbohydrates. Increases in microbial biomass and fermentation products alter the rumen’s structure and function (Alipour et al., 2018; Drackley, 2008). Around weaning stage, a fully functional rumen and adult-like microbiota are established (Lallès, 2012). Additionally, in humans, the gut microbiota plays a critical role in the development and differentiation of the intestinal lumen lining epithelial cells as well as the immune system’s homeostatic maintenance, which includes tolerance to dietary antigens (Guarner and Malagelada, 2003).

## 5 The role of microbiota in reproductive health

### 5.1 Male reproductive efficiency

Since the testis cannot synthesize nutrients, the gut microbiota assists the testis by metabolizing nutrients. The primary modulator of mammalian bone mass is the gut microbiota, which controls the conversion of blood to bone calcium and, consequently, Ca2+ levels in the reproductive system. *Bifidobacteria* and *Lactobacillus* in genetically modified organisms influence the intake of calcium from food. By lowering the pH of the intestine, SCFAs decrease the production of calcium phosphate and increase calcium absorption (D’Amelio and Sassi, 2017). A crucial component of fertilization in mammal is calcium; as it controls sperm motility, which directly affects the likelihood of sperm-egg fusion. The activation of calcium ion channels on the sperm flagellum is essential for facilitating sperm motility into the female reproductive tract, a phenomenon referred to as sperm capacitation (Vyklicka and Lishko, 2020). Folic acid primarily originates from bacterial metabolites and dietary supplements. Proton-coupled folate transporter in colon cells absorbs GTP, erythrose 4-phosphate, and phosphoenolpyruvate to generate tetrahydrofolic acid (THFA), which is then distributed throughout the body via the circulatory system. Genomic analysis has identified various bacteria, including *Salmonella enterica* (Proteobacteria), *Bifidobacterium* spp., (Actinobacteria), *Fusobacterium varium* (Fusobacteria), *Clostridium difficile*, *Lactobacillus plantarum*, *L. reuteri*, *L. delbrueckii* ssp., *Bulgaricus*, and *Streptococcus thermophilus* (Firmicutes), as contributors to THFA synthesis (Yoshii et al., 2019). Intake of folic acid improves semen quality and structural integrity of testicular tissue, especially when animals exposed to reproductive toxins. Folic acid plays a protective role in supporting germ cells against oxidative stress and inflammation, preventing DNA damage and apoptosis. It also protects germ cells from oxidative stress, allowing them to develop and differentiate (Cai et al., 2022). Furthermore, the altered composition of the gut microbiota, including its metabolites, endotoxins, and pro-inflammatory substances, has the potential to affect gut permeability and immune function, can adversely affect the reproductive system and the immune environment of the testis (Guo et al., 2020). The gut microbiome, considered an endocrine organ, impacts the reproductive endocrine system through sex hormone fluctuations (Ashonibare et al., 2024). The amount of testosterone in the blood can also be altered by the gut microbiome (Qi et al., 2021). The gut microbiota has been identified as a key regulator of androgen production and metabolism. By producing enzymes, the gut microbiota can generate and convert androgens, actively partaking in microbial processes that break down testosterone (Lv et al., 2024). For example, *Clostridium scindens* exhibits a high potential for converting glucocorticoids into androgens, while certain proteobacteria possess the ability to degrade androgen. These intricate interactions between gut microbes and androgen metabolism significantly update our understanding of male reproduction (Emenike et al., 2023; Wang et al., 2014; Yang et al., 2016).

### 5.2 Female reproductive efficiency

The female reproductive tract is home to a diverse ecosystem of chemicals, immune components, host cells, and microbes. The complex interactions that occur among bacteria, immune cells, and host cells within the female reproductive system help to maintain reproductive tract homeostasis (Gholiof et al., 2022). The gut microbiome, which is considered an extended endocrine organ, plays an important role in female reproductive health (Chadchan et al., 2022). According to microbiome’s evaluations, the vaginal microbiota accounts for around 9% of the overall human microbiome (Saraf et al., 2021). The bacterial genera like *Prevotella*, *Bifidobacterium*, *Gardnerella*, *Atopobium*, *Megasphaera*, *Sneathia*, and *Anaerococcus* are associated with various reproductive stages, including gamete development, fertilization, the initiation and preservation of pregnancy, and the microbial colonization of the developing fetus or infant (D’Argenio, 2018; Franasiak and Scott, 2015; Moreno and Simon, 2018).The secretion of *β*-glucuronidase can be modulated by the gut microbiota, which is crucial and impact the estrogen levels. Dysbiosis or decrease in the diversity of the gut microbiota can cause fluctuations the estrogen levels in blood and β-glucuronidase activity. These variations can contribute to obesity, metabolic syndrome, cancer, endometrial hyperplasia, endometriosis, PCOS, and infertility (Baker et al., 2017; Chadchan et al., 2022). As demonstrated in Table 2, the composition of gut microbiota varies significantly across different mammalian species, with notable differences in key bacterial phyla that have been linked to reproductive health outcomes.

**Table 2:** A comparative analysis of the microbiota across diverse mammalian species and its influence on reproductive processes.

Aspect	Human	Non-human	Rate	Cattle, Sheep	Horse
Vaginal microbiota	*Lactobacillus* species dominate, with acidic pH aiding infection defense, while disruptions like bacterial vaginosis affect fertility (Olson et al., 2018).	A more diverse microbiota, less dependent on Lactobacillus, relies on immune adaptations for infection defense (Nuriel-Ohayon et al., 2016).	The vaginal microbiome shifts significantly during the estrous cycle, with reduced Lactobacillus dominance and a greater influence on mating behaviors (Miller et al., 2016).	*Lactobacillus* is less prevalent, with microbial shifts influenced by reproductive cycles. Infections like metritis reduce fertility (Santos and Bicalho, 2012).	In marsupials, pouch microbiota varies with reproductive architecture, while in horses, vaginal microbiome diversity impacts fertility (Chhour et al., 2010).
Seminal microbiota	A diverse microbiome influences sperm motility, with an overgrowth of bacteria like Enterococcus associated with male infertility (Jendraszak et al., 2024).	The seminal microbiome in non-human primates affects sperm quality, but it is less studied than in humans (Camargo et al., 2017).	Microbial imbalances in seminal fluid are less studied but can similarly affect sperm motility and reproductive success, as in humans (Bicalho et al., 2017).	The seminal microbiome influences sperm quality in animals, with homogeneous compositions linked to higher fertility (Castillo et al., 2015).	Microbial imbalances in horse seminal fluid can impair sperm motility and fertility, despite a diverse seminal microbiota composition (Al-Essawe et al., 2018).
Microbial changes during pregnancy	As gastrointestinal diversity decreases, *Lactobacillus* dominance in the vaginal microbiome rises. Dysbiosis may lead to preterm birth and preeclampsia (Koren, Goodrich, Cullender, Spor, Laitinen, Bäckhed, et al., 2012).	The vaginal microbiota in pregnancy changes more subtly than in humans, relying on immune system regulation (Weichhart et al., 2015).	Gut and vaginal microbiota shifts during pregnancy facilitate microbial transfer to the child, influencing immune system development (Rautava et al., 2012).	Pregnancy has a smaller impact on livestock microbiota, but reproductive diseases like metritis can be detrimental (Liu et al., 2022).	Marsupials experience unique microbial changes due to the pouch environment, while in horses, microbial stability during pregnancy is crucial for fetal health (Hand et al., 2016).
Microbial transfer to offspring	Vaginal birth introduces beneficial bacteria to newborns, and breastfeeding offers additional microbial exposure, crucial for immune development (Dominguez-Bello et al., 2010).	Similar to humans, though with different bacterial species and less *Lactobacillus* dominance, breastfeeding still transfers beneficial bacteria (Łubiech and Twarużek, 2020).	Vaginal delivery and breastfeeding support early microbial colonization, aiding the development of the newborn’s immune system (Bäckhed et al., 2015a).	Vaginal delivery and colostrum transfer crucial microorganisms for infant survival, while microbial diversity supports immune priming (Reynolds and Bettini, 2023).	In marsupials, exposure to pouch microbiota is vital for offspring survival, while in horses, similar microbial transfer occurs during birth and nursing (Zhong and Zhong, 2016).
Reproductive cycle and microbial shifts	The microbiota remains largely stable throughout the reproductive cycle, except for pregnancy-related changes that protect the fetus and support reproductive health (Borody and Khoruts, 2011).	Hormonal changes during the reproductive cycle significantly alter microbial composition, directly affecting reproductive success (Antwis et al., 2019).	Microbial composition shifts with the estrous cycle, affecting reproductive behaviors and outcomes (Qi et al., 2021a).	Microbial shifts during the menstrual cycle enhance fertility and help prevent diseases like metritis and vaginitis (Molina et al., 2020).	Microbial changes in seasonal breeders like horses align with hormonal shifts, boosting reproductive success and supporting pregnancy (Yatsunenko et al., 2012).
Impact of dysbiosis on reproduction	Dysbiosis is linked to infertility, premature birth, and bacterial vaginosis. In men, seminal microbiota imbalances reduce sperm motility (Baker et al., 2018).	Dysbiosis leads to reproductive disorders like infertility, though research in this area is less advanced compared to human studies (Markle et al., 2013).	Dysbiosis affects fertility and pregnancy outcomes by disrupting reproductive health and immune system regulation (Morgan, 2015).	Dysbiosis leads to reproductive diseases like metritis, mastitis, and vaginitis, significantly lowering reproductive success (Bicalho and Oikonomou, 2013).	Dysbiosis in marsupials can disrupt pouch microbiota, while microbial imbalances in horses are linked to reduced fertility and reproductive issues (Garcia-Garcia et al., 2022).

### 5.3 Mother-newborn bond

The mother’s oral, stomach, and vaginal microbiota are fluctuating throughout pregnancy. These changes are caused by a variety of factors, including host genes, antibiotic use, infections, diet, and stress (Codagnone et al., 2019; Goodrich et al., 2016; Kim et al., 2017; Zhou et al., 2020). According to Romero et al.’s (2014) study, a large number of *Lactobacillus* spp., were discovered among other vaginal microbiota in healthy pregnant women, and this species is more stable in these women than in nonpregnant healthy women. About 90% of *bifidobacteria* and were found in the microbiota of breastfed infants, whereas 40–60% were found in formula-fed infants. Furthermore, breastfed newborns develop their gut microbiota more quickly than infants nourished via formula milk. Furthermore, studies indicated microbial variations between breastfed and non-breastfed infants, with *Bifidobacterium adolescentis* colonization being more common in the breastfed newborn and *Bifidobacterium catenulatum* lacking (Coppa et al., 2011).

## 6 Biological association and implications for human and veterinary medicine

### 6.1 Gut-brain axis

Gut microbes actively contribute to neurodevelopmental processes, including the formation of the blood–brain barrier, neurogenesis, microglial maturation, and myelination (Cerdó et al., 2020; Parker et al., 2020). The gut-brain axis is a bidirectional communication network connecting the intestinal and central nervous systems. This network extends beyond anatomical connections to include endocrine, humoral, metabolic, and immunological pathways. The autonomic nervous system, hypothalamic–pituitary–adrenal (HPA) axis, and gastrointestinal (GI) nerves form key links between the gut and brain, enabling the brain to regulate intestinal functions, such as immune cell activity, while allowing the gut to impact mood, cognition, and mental health. Ongoing research is uncovering the mechanisms by which microbiota influence the brain’s emotional and cognitive centers, both directly and indirectly. Studies have demonstrated that fluctuations in microbiota are associated with alterations in these communication systems (Mayer et al., 2014). Additionally, research suggests that the composition of the gut microbiota may play a role in influencing fetal and neonatal brain development (Douglas-Escobar et al., 2013). Bacterial metabolites, particularly short-chain fatty acids (SCFAs) produced by the fermentation of dietary carbohydrates, act as key humoral modulators. These microbiota-derived SCFAs can cross the blood–brain barrier and have been shown to regulate microglial homeostasis, which is crucial for proper brain development, tissue maintenance, and behavioral modulation (Mayer et al., 2015). SCFAs regulate the secretion of gut peptides from enteroendocrine cells and influence the production of gut-derived serotonin by enterochromaffin cells. Both processes play a crucial role in modulating gut-brain hormonal communication (Wang and Kasper, 2014). The gut produces approximately 95% of the body’s total serotonin, with most of it found in plasma. While serotonin has key roles in gut function and peripheral metabolism, it can also locally activate afferent nerve terminals that connect directly to the central nervous system (Macfabe, 2013). Treatment with *Lactobacillus rhamnosus* reduced stress-induced corticosterone levels and alleviated anxiety- and depression-related behaviors in rats. Notably, in mice that underwent vagal nerve dissection, no neurochemical or behavioral changes were observed, confirming that the vagus nerve is the primary pathway for communication between gut bacteria and the brain (Kim and Shim, 2023). A balanced gut microbiota plays a crucial role, both directly and indirectly, in maintaining the environment necessary for optimal neural development (Sarubbo et al., 2022). A recent comparative study of germ-free (GF) and specific pathogen-free (SPF) mice has identified several gut microbial compounds that can cross the placenta and enter the fetal compartment, where they influence and regulate prenatal developmental processes (Pessa-Morikawa et al., 2022). Tail biting is a prevalent and harmful issue in intensive pig farming. A recent study estimated that tail biting in pigs could reduce net profit by up to USD 23.00 per pig, leading to annual losses amounting to millions of dollars for the pork industry (Henry et al., 2021). Regarding tail biting and the porcine microbiome, no significant difference in alpha diversity was observed between tail-biters and the control group. However, a consistent difference in beta diversity was noted among tail-biters, victims, and the control groups (Verbeek et al., 2021). In pigs exhibiting tail-biting behavior, victims of tail-biting, and those showing other anxiety-related behaviors, certain Firmicutes families and orders, specifically Clostridiales (including *Ruminococcocus*, Lachnospiraceae, and Clostridiales Family XII), showed a relative increase in abundance. In contrast, other Firmicutes, particularly Lactobacillus spp., exhibited a relative decrease in abundance. The composition and abundance of gut microbiota, particularly Firmicutes and Bacteroidetes, have been linked to various mental disorders in humans, including anxiety, depression, bipolar disorder, autism spectrum disorder (ASD), and schizophrenia (Xiong et al., 2023).

### 6.2 Biological association between microbiota and host

Numerous physiological function of host, such as nutritional and metabolic processes (Degnan et al., 2014), immune system modulation and regulation (Francino, 2014), and brain and behavior development (Hsiao et al., 2013), are associated with the microbiota. According to recent research, the host and normal microbiota interact in the following four ways: firstly, the microbiota acts as a barrier against pathogens; secondly, it modifies the host mucosa’s permeability; thirdly, it influences the host’s capability to extract energy from food and use it metabolically; and lastly, it influences the immune system. By these four ways of interaction also increase the host’s susceptibility to diseases when the normal microbiota is altered. For instance, commensal bacteria exist in the outer mucus layer epithelial tissues, therefore changes in the normal microbiota affect intestinal mucosa permeability. When the body is healthy, the inner mucus coating works as a physical barrier, preventing bacteria from making direct contact with the epithelial layer (Hertli and Zimmermann, 2022). Intestinal permeability dysfunction can result from disruption of mucosal development, such as through dysbiosis, and has been linked to an inclination toward immunological disorders (Groschwitz and Hogan, 2009). Metabolites from various commensal bacteria, such as *Bifidobacterium lactis*, *Bacteroides fragilis* and *Akkermansia muciniphila* (Lindfors et al., 2008; Hsiao et al., 2013; Chelakkot et al., 2018; Everard et al., 2013; Plovier et al., 2016), affect mucin production and keep tight junction appearance to maintain the intestinal barrier.

**Figure 4:**
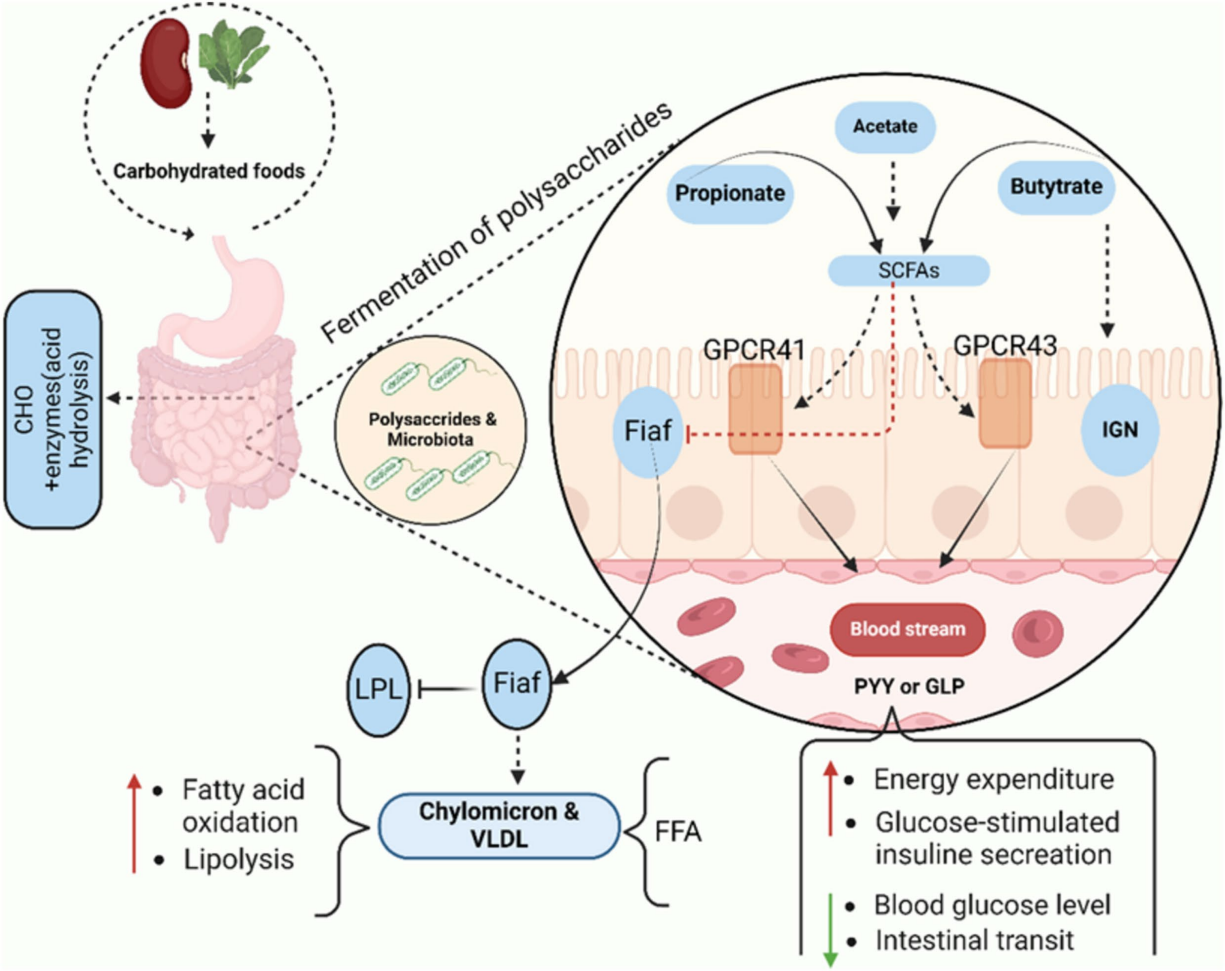
This pathway explores the mechanisms underlying metabolic interactions between host and microbiota. Fermentation of polysaccharides where microorganisms convert these sugars into various byproducts, including short-chain fatty acids (SCFAs) like acetate, propionate, and butyrate. Propionate (Free Fatty Acid Receptor 3, FFAR3) is a primary ligand for GPR41 and activates GPR41. The GPR41 can influence a variety of cellular responses, including hormone release like peptide YY, and increase energy expenditure while decreasing gastrointestinal motility. Acetate and propionate are ligands for GPR43 (Free Fatty Acid Receptor 2, FFAR2) and lead to the release of hormones such as glucagon-like peptide-1 (GLP-1), affecting insulin secretion and glucose homeostasis. Butytrate works on Intestinal gluconeogenesis (IGN) thus regulate energy homeostasis. Fiaf (Fasting-Induced Adipose Factor) which inhibits lipoprotein lipase (LPL), and releases fatty acids from circulating chylomicrons and VLDL lead to promotes fatty acid oxidation and lipolysis (breakdown of stored fat). SCFAs act as metabolic signals that influence various pathways, including fat metabolism, insulin secretion, and gut motility. Also, this interaction offers insights into therapeutic strategies for metabolic disorders such as obesity, diabetes, and dyslipidemia, by modulating gut microbiota or SCFA production. Created with BioRender.com.

Regarding the third point, the gut microbiota has determined the bioavailability of vitamins derived from food. A good example is the conversion of vitamin K1 to vitamin K2, which is assisted by commensal bacteria such as *Veillonella*, *Eubacterium lentum*, *Enterobacter*, and *Bacteroides* (Biesalski, 2016; Blacher et al., 2017). Vitamin K acts as a cofactor to regulate the coagulation cascade and immunological processes in mammalian species. The presence of K2 may lower the incidence of osteoporosis and coronary heart disease (Beulens et al., 2013). The body absorbs tryptophan (Trp), an essential amino acid, through food. The intestinal microbiota has the ability to metabolize it and produce aryl hydrocarbon receptor (AhR) ligands. The body’s various cells express AhR, which affects a number of host immunological responses and pathways, such as the cell cycle, immune system, neurological signaling, and reactions to xenobiotics, antioxidants, and hormone-like estrogen (Agus et al., 2018). AhR ligands regulate the growth activity, and synthesis of metabolites in addition to mucosal immune cells. Tryptophanase is a bacterial enzyme and the following group of *Lactobacilli* (Lamas et al., 2018), *Peptostreptococcus russellii* (Wlodarska et al., 2017), and *E. coli* (Agus et al., 2018; Alexeev et al., 2018) use it for converting tryptophan into indole. Whereas, indole stimulates the synthesis of IL-22, which improves intestinal homeostasis (Agus et al., 2018; Alexeev et al., 2018; Lamas et al., 2018). Multiple cytokines influence intestinal epithelial cells, increasing proliferation and the generation of antimicrobial peptides (Lamas et al., 2018). Furthermore, AhR ligands reduce the possibility of intestinal pathogen colonization. *In-vitro* experiment showed that indole-3-acetonitrile prevents the *Candida albicans* multiplication in the growing biofilms and adhering to gut epithelial cells (Oh et al., 2014). The lower production of these ligands by the microbiota in IBD patients may be linked to the decreased production of AhRs by the immune cells like (CD3+, CD4+, CD56+, and CD25+) (Lamas et al., 2018). Moreover, interferon (IFN)-*γ* secretion is decreased and IL-22 production is increased by AhR ligands, which counteract inflammatory responses (Monteleone et al., 2011). In the future, therapies for autoimmune disorders, infections, and chronic inflammation may be developed by focusing on the IL-22 pathway. *Bifidobacterium infantis* activates the rate-limiting enzyme like indoleamine 2,3-dioxygenase-1 (IDO-1) for the conversion of Trp to kynurenic acid. This molecule is crucial for both immunological responses and neuronal processes. Experiments in rats have shown that *Bifidobacterium infantis* may have an antidepressant effect (Tian et al., 2019). Furthermore, the microbiota can convert Trp into indole, promoting colonic L cells to generate GLP-1 and perhaps contributing to the genesis of metabolic syndrome (Chimerel et al., 2014). Tryptophan hydroxylase is an enzyme that converts Trp to tryptamine, and it is activated by *Lactobacillus bulgaricus*, *Clostridium sporogenes*, and *Ruminococcus gnavus* (Williams et al., 2014). Tryptamine increases the inhibitory response of cells to serotonin by binding to trace amine-associated receptors in the brain (Williams et al., 2014). Tryptamine also attaches itself to the sigma-2 receptor in mice, which may play a role in the onset of Alzheimer’s and cancer (Williams et al., 2014; Yang et al., 2020). Tryptamine causes enterochromaffin cells in the gastrointestinal tract to secrete more serotonin. Variations in intestinal serotonin levels are thought to affect intestinal motility and may contribute to the pathophysiology of inflammatory bowel disease (Williams et al., 2014). Tempering serotonin receptors may aid in the treatment of irritable bowel syndrome (IBS) because serotonin is an important neurotransmitter for signaling in the enteric nervous system (Williams et al., 2014). Carbohydrate metabolism pathways are investigated to determine the fundamental processes of host-microbiome metabolic interactions. Microbes undergo fermentation, a metabolic process that converts sugars into several byproducts, including butyrate, propionate, and acetate, which are classified as short-chain fatty acids (SCFAs) (Jansma and El Aidy, 2021). SCFAs, in particular, have a significant impact on host metabolism, immunological function by providing energy to gut epithelial cells and to beneficial bacteria, gut barrier function, gut cell proliferation, and even gut-brain axis communication (Shtossel et al., 2024). The primary ligand for GPR41 (Free Fatty Acid Receptor 3, or FFAR3), binds to propionate and activates GPR41. Propionate-induced GPR41 activation can control a number of cellular reactions, including the release of hormones like peptide YY (PYY), which lowers gut motility and increases energy expenditure. GPR43 (Free Fatty Acid Receptor 2, FFAR2) ligands such as acetate and propionate can cause the release of hormones like glucagon-like peptide-1 (GLP-1), which can have an impact on insulin secretion and glucose homeostasis while the intestinal gluconeogenesis (IGN), butyrate has beneficial effects on glucose and energy homeostasis. This complex interplay emphasizes how crucial the gut microbiota and its metabolites are to preserving a healthy and mutually beneficial relationship with the host and shown in (Figure 4).

### 6.3 Biological association between pathogen and host microbiota

Animals live in symbiosis with numerous microbial species and it is widely recognized that host microbiota help to prevent or fight infection (Britton and Young, 2014; Chiu et al., 2017; Lamousé-Smith et al., 2021; Shanahan, 2010), as like changes in the microbial landscape, as well as microbiota components, have the potential to exacerbate infections and disease severity. Pathogens and pathobionts can cause opportunistic infection by exploiting microbiota metabolites or taking advantage of a host’s defenses being depleted and changing environment. The microbiota may potentially support a more virulent evolutionary pathway for invading diseases (Stevens et al., 2021). It has shown that the emergence of pathogenic bacterial species and disruption of the gut microbial community, known as dysbiosis, are linked to the development of a number of systemic illnesses, including autoimmune diseases (Mousa et al., 2022). The host microbiota serves as a pathogen barrier or deterrent by acting through innate immune hubs, has the ability to prevent or alleviate an increase in inflammation. Various components, such as epitheloid cells’ microbiota, innate lymphocytes (Han et al., 2013), and adaptive lymphocytes (Kubinak et al., 2015), all interact with pattern recognition receptors. Noteworthy examples include the modulation of goblet cell functions (Wang et al., 2015) and the control of granulopoiesis through Myd88/TICAM by microbiota-released substances (Balmer et al., 2014). Related to changes the microbial landscape, intestinal microbiota barrier function is exemplified by *Clostridium difficile* infection (CDI). When a healthy microbiota is lost, conditions arise that facilitate the infection and disease-causing potential of *C. difficile*. Antibiotic-induced dysbiosis (abnormal microbiota) and subsequent *Clostridioides difficile* infection (CDI) have been linked to alterations in epithelial permeability, TH17 function, and TLR signaling. This strong evidence from both human and veterinary studies demonstrates that restoring the microbiota through fecal microbiota transplantation (FMT) resolves Clostridioides difficile infection (CDI) and increases resistance to recurrence, lending credence to the theory that changes in the microbiota cause CDI. In these studies, fecal transplants were administered either orally or rectally, transferring healthy donor microbiota into the gastrointestinal tract of affected animals, effectively re-establishing microbial balance and suppressing pathogenic bacteria (Barbara et al., 2021).This approach has shown promise in veterinary medicine, particularly in cases of chronic gastrointestinal diseases.

Microbiota metabolites have a diverse impact in host health, including priming the immune structure, acting as antimicrobials, and aiding host metabolism (McCarville et al., 2020; Rooks and Garrett, 2016). However, the same metabolites may also serve as a food source for occupying pathogens. Metabolic interactions create new environments that could potentially support pathogens, boosting their ability to produce energy and become more virulent (San Roman and Wagner, 2018). For instance, the gut bacteria *Bacteroides thetaiotaomicron* can worsen infections caused by enterohaemorrhagic *Escherichia coli* through metabolic interactions (Curtis et al., 2014). Unique microbial populations influence the outcomes of infections by producing different metabolites. The intricate nature of these interactions poses a challenge in microbiome research.

## 7 The modeling approaches used to study the microbiota

### 7.1 Germ-free animals as crucial models for investigating host-microbial interactions

Valid experimental models for examining the host-microbial interactions in health, disorders, and diseases are germ-free (GF) animals (Al-Asmakh and Zadjali, 2015; Bhattarai and Kashyap, 2016). While mice are commonly employed in germ-free models, other species, such as zebrafish, may also be utilized in these investigations (Melancon et al., 2017). For a variety of research projects, germ-free pigs, chickens, and dogs were also raised (Harding et al., 2010). Numerous fields, including cancer therapy, metabolism, diabetes, reproduction, cardiovascular issues, and bone homeostasis, can benefit from its application. Recently, the significance of the gut-brain axis in the brain development of mammals has been established (Diaz Heijtz et al., 2011). Mice, in particular, have been used in a number of germ-free animal studies on behavioral and brain disorders or diseases, including anxiety, depression, schizophrenia, and autism (Horne and Foster, 2018; Neufeld et al., 2010). Consequently, GF animals are an appropriate and accessible model for research on the gut-brain axis. It is feasible to develop genetically modified mice as GF to investigate the interactions between specific genes and the gut microbiota. The phylogenetic composition of the human microbiota can be recapitalized through the inoculation of human gut microbiota into humanized gnotobiotic GF mice. These models serve as effective tools for better understanding the composition of gut microbiota in systems that resemble humans (Al-Asmakh and Zadjali, 2015). Mice of various breeds can also be utilized as a germ-free model. For instance, GF models have been utilized to study anxiety and type 2 diabetes in Swiss-Webster and C57BL/6 mice, respectively (Hansen et al., 2014). In animals given antibiotics, phenotypic transfers through microbial transplantation are also feasible. The most reliable controlled models for microbial transplantation appear to be GF animals (Croswell et al., 2009).

GF animals are used as models in preclinical research to better understand the impact of bacteria on host development and function, rather than to replicate human conditions. Furthermore, we were able to ascertain the influence of a particular strain of bacteria on various health-related problems by colonizing the animals with either a single strain or a combination of known strains. There are a few substitutes for the germ-free murine model. Numerous approaches have been investigated as substitutes for the germ-free (GF) murine model, including probiotic diets, fecal transplants, antibiotic therapy, and humanization of mice by colonizing them with human microbiota. These methods aim to manipulate the gut microbiota and study its effects on host physiology. Fecal microbiota transplants (FMT) involve transferring microbiota from healthy donors into germ-free or antibiotic-treated mice, while humanized mouse models are generated by colonizing GF mice with human fecal material to mimic the human microbiome. Although challenges exist in extrapolating results from mouse models to humans due to differences in microbiota composition, GF animals remain the most effective alternative to date for studying microbiota-related health outcomes, particularly in the context of host-microbiota interactions (Gabay et al., 2020).

### 7.2 Interactions through mono and bi-associated gnotobiology models

The study of particular microbial species or strains that colonize in GF animals is known as gnotobiology. Research involving animals with a single or pair of commensal species allows us to study how microbes affect their hosts in a simple ecosystem. Mono-associated animal models can help us understand the microbe’s niche, resources, and host response. Likewise, studies on bi-associated animals can shed light on the interactions that occur between a pair of microbes and their host, as well as whether the competition that occurs during colonization for resources and space which affects the functional roles that the microbes establish within the gut ecosystem (Bäckhed et al., 2005; Ley et al., 2008; Sadeghi et al., 2023). The fecal models of *Bacteroides thetaiotaomicron* and *Eubacterium rectale* developed by Mahowald (Mahowald et al., 2009) demonstrated that both organisms undergo a significant shift in gene expression patterns when transitioning from mono-association (where each organism was introduced individually into germ-free mice) to co-colonization (where both organisms were introduced together). This shift was identified through comparative transcriptomic analyses, which revealed how these bacteria respond differently to their environment when colonizing the gut in the presence of other microbial species. The study highlights the complex interactions between gut microbes and their adaptive behavior within microbial communities. This is because the variation in the gut microbiome is determined by the composition of its members and the sequence in which they colonize each other. These models are useful for understanding the specific niches occupied by individual members of the gut microbiota. However, they are further complicated by the fact that these bacteria respond differently to co-colonization based on species and sequence. For example, in newborn human babies, the obligate anaerobes take over the GIT after the facultative anaerobes (Mahowald et al., 2009). Once we start introducing GF animals to the world, it’s not always clear how their interactions with different commensal microbes will play out. It’s not always easy to predict how they’ll behave in a natural state.

### 7.3 The altered schaedler flora: a consistent model for investigating gut ecosystems

Russell W. Schaedler introduced the idea of multiple-associated animal models in the middle of the 1960s (Dewhirst et al., 1999). The aim was to create a standardized gut microbiota to serve as a reliable research tool. This was accomplished by intentionally populating GF mice with eight specific bacterial strains derived from conventional mice. The widely utilized standardized poly-associated flora in current laboratory settings is the Altered Schaedler Flora (ASF), a modification of the original Schaedler Flora dating back to 1978. The ASF, which includes four fusiform bacteria that are highly sensitive to oxygen (EOS), two *Lactobacillus* spp., a cousin of *Bacteroides distasonis*, and a spiral-shaped bacterium from the Flexistripes phylum, was recently characterized using 16S rRNA profiling (Dewhirst et al., 1999). It is increasingly important for this set of standardized flora to accurately mimic the ecosystem of gut of a conventional animal, rather than focusing solely on the specific identities of the bacterial strains constituting the ASF. Fifty percent of the bacterial strains present in the ASF consist of *Firmicutes* EOS bacteria. Multiple recent studies examining the gut microenvironment consistently reveal that EOS bacteria make up the predominant portion of the gastrointestinal microbiota in mice and rats, surpassing aerobes by a ratio of at least 1,000:1 (Savage, 1970), and facultative anaerobes by a ratio of 100:1. Two of the ASF’s members are Lactobacillus spp., an aerotolerant subgroup of Firmicutes that frequently colonize human stomachs and small intestines as well as other conventional vertebrate mammals (Roach et al., 1977). Because ASF animal models are colonized with a specific set of intestinal flora, they are thought to be beneficial. Animal shelters differ significantly in terms of their flora, as do cages within the same facility. Therefore, by reducing variability arising from variations in the gut ecosystems of different research animals, the colonization of animals with the standardized ASF flora enhances cross-study comparability. ASF has been successfully introduced into new mouse strains as knockout strains and transgenics through embryo transfer. According to recent 16S rRNA sequencing of the mouse gut flora, the ASF in a mouse colony is stable over an extended period of time (i.e., free of contaminating bacteria) (Stehr et al., 2009). These stability tests do not, however, remove the necessity of actively monitoring the ASF’s composition, as unnoticed changes in the flora may materially impact the results of later experiments. Using a consistent gut flora in ASF animal models is beneficial. However, the intricacy of the typical intestinal microbiota, which includes around 800 to 1,000 bacterial species, cannot be accurately mimicked by an intestinal microbiota consisting of only eight bacterial species (Bäckhed et al., 2005). The ASF can accurately represent the dominant phyla found in a conventional vertebrate animal, but it is not expected to show host-microbiome interactions that are entirely comparable to those found in a natural gut ecosystem because so few organisms can replicate its community dynamics.

## 8 Conclusion and future implications for practice

In conclusion, we investigate the unique microbiota of the oral, respiratory, skin, gut, and genital tracts, emphasizing their individual roles and cumulative impact on mammalian host health. Also, highlights the pivotal role of gut microbiota in regulating health, production, and reproduction in both humans and animals, influencing key metabolic, immune, and reproductive processes. Understanding these intricate host-microbiota interactions has profound implications across life cycle, host signaling pathways. Finally, authors investigate into modeling approaches for microbiota research, including germ-free animal models, mono-associated and bi-associated models, and poly-associated animal models. These models provide essential tools for studying the dynamic nature of microbial communities and their effects on host organisms. Translationally, the findings suggest that microbiota-based therapies hold promise for enhancing overall health and productivity in agricultural species, as well as improving reproductive health in clinical applications. Despite significant progress in understanding gut microbiota’s role in mammalian health, production, and reproduction, several gaps remain. Causality between specific microbial communities and host outcomes is difficult to establish, with most studies relying on correlative data. Research has primarily focused on model organisms, limiting broader application to diverse species. Environmental, dietary, and geographic variations also complicate universal conclusions. Additionally, most studies examine short-term effects, underscoring the need for more longitudinal research to understand the sustained impact of microbiota on host physiology. Addressing these gaps will enhance the development of microbiota-based interventions.

Implications for practice

- Livestock management

Modulating the gut microbiota through the use of prebiotics, probiotics, or optimized feed strategies can enhance digestion and nutrient absorption. This approach may lead to improved feed efficiency, reduced feed costs, and accelerated growth rates.

Maintaining a balanced gut microbiota plays a crucial role in enhancing immune function and reducing the risk of disease. Probiotic supplements and fecal microbiota transplantation (FMT) present promising alternatives to antibiotics for disease prevention.

Farmers can reduce their reliance on antibiotics by promoting a healthy microbiome in livestock, which helps mitigate the risk of antibiotic resistance in animals and the human food chain.

Animals with a balanced microbiota may produce higher-quality meat and dairy products, potentially enhancing taste, texture, and nutritional value.

- Veterinary practices

Veterinarians can design more precise and individualized treatments for animals by analyzing the composition of their microbiome. This tailored approach may enhance therapeutic outcomes for diseases associated with microbial dysbiosis, such as gastrointestinal disorders, dermatological infections, and respiratory conditions.

The analysis of microbiota composition can facilitate early diagnosis of certain diseases, enabling more effective and timely interventions. This approach is particularly advantageous for detecting subclinical infections and identifying animals at risk for metabolic or inflammatory conditions.

Probiotics, synbiotics, and fecal microbiota transplantation (FMT) can be integrated into routine veterinary care, offering less invasive alternatives for managing chronic conditions. These approaches may reduce the reliance on pharmaceutical medications while promoting long-term health in animals.

- Public health

Regulating cattle microbiota can help reduce the prevalence of harmful pathogens, such as Salmonella, Campylobacter, and *E. coli*, in the food supply, thereby decreasing the risk of foodborne illnesses.

Reducing antibiotic use in livestock through microbiota management directly addresses the escalating public health threat of antibiotic-resistant microorganisms. Livestock serve as a significant reservoir for resistant pathogens, which can be transmitted to humans through food, water, or direct contact.

A deeper understanding of animal microbiota can contribute to the prevention of zoonotic diseases, such as avian influenza and coronaviruses, by identifying and managing microbial factors that facilitate pathogen transmission between animals and humans.

Microbiota regulation in cattle may help reduce the environmental impact of livestock farming. Animals with a healthier and more balanced gut microbiota tend to produce lower methane emissions, a potent greenhouse gas, and require fewer resources for feeding and maintenance.
